# Tuning Organic Carbon Dioxide Absorbents for Carbonation and Decarbonation

**DOI:** 10.1038/srep10688

**Published:** 2015-06-02

**Authors:** Ramachandran Rajamanickam, Hyungsoo Kim, Ji-Woong Park

**Affiliations:** 1 School of Materials Science and Engineering, Gwangju Institute of Science and Technology, Gwangju, South Korea, 500-712

## Abstract

The reaction of carbon dioxide with a mixture of a superbase and alcohol affords a superbase alkylcarbonate salt via a process that can be reversed at elevated temperatures. To utilize the unique chemistry of superbases for carbon capture technology, it is essential to facilitate carbonation and decarbonation at desired temperatures in an easily controllable manner. Here, we demonstrate that the thermal stabilities of the alkylcarbonate salts of superbases in organic solutions can be tuned by adjusting the compositions of hydroxylic solvent and polar aprotic solvent mixtures, thereby enabling the best possible performances to be obtained from the various carbon dioxide capture agents based on these materials. The findings provides valuable insights into the design and optimization of organic carbon dioxide absorbents.

Numerous approaches to capturing CO_2_ have been used to reduce its emission into the atmosphere^1^,^2^,^3^,^4^,^5^,^6^,^7^,^8^,^9^. The most well-established technology for capturing CO_2_ is based on aqueous amine solutions^10^; however, improved sorbent systems that can significantly reduce the energy cost of regeneration are in great demand. Non-aqueous absorbent systems such as ionic liquids or alcoholic solutions of superbases or amines are promising candidates because of their low heat capacity^11^,^12^,^13^,^14^,^15^. It is also possible to exclude latent heat from the regeneration energy by using high boiling point solvents.

The carbonation of the organic absorbents, regardless whether their starting form is neutral or ionic liquids, produces ion pairs that are stable in solutions at the CO_2_ capture conditions. Different forms of capture agents have been reported to show high gravimetric absorption of CO_2_^16^,^17^,^18^,^19^,^20^,^21^. In most of the previous studies, however, the optimal conditions to revert the ion pairs to the original absorbents have not been sought seriously. The most frequently used method is flushing the carbonated solution with inert gas at elevated temperature, which reproduces a mixed gas that again needs separation. It is essential to develop an efficient method to control the temperature of carbonation and decarbonation of the organic solutions. The temperature tunability of the organic CO_2_-absorbing solutions will enable optimization of the system for low energy and low viscosity as well as high capacity carbon capture.

The thermal stabilities of ionic species are greatly influenced by the ion-solvating ability of the solvents. In general, ionic solutes are solvated in distinct manners by protic and aprotic polar solvents^22^,^23^. While the anions are solvated efficiently by protic solvents via hydrogen bonding, they are poorly solvated by aprotic solvents. On the other hand, the cations are well solvated by polar aprotic solvents^22^,^24^. The poor solvation of anions in a polar aprotic solvent shifts the acid–base equilibrium of organic acids in favor of neutral forms, resulting in higher pK_a_ values than those found for aqueous solutions^25^,^26^. We hypothesize that these general effects of organic solvents play a significant role in the stability of the carbonate salt ion pairs that form by carbonizing the organic solution mixture.

It has been reported that the decarbonation temperature of a carbonated alkanolguanidine salt is decreased in their biphasic mixture with a non-polar solvent (such as *n*-alkane)^27^. While this demonstrates that utilizing solvent effect is a promising approach for attaining the tunability of decarbonation temperatures for the organic CO_2_ absorbents, it is yet to understand the critical factor that controls the thermal stability of the organic carbonated salts in solutions. In addition, it is advantageous for process development if the carbonated solution can form a homogenous solution rather than the biphasic mixture.

Here we show that ternary mixtures consisting of a superbase, hydroxylic solvent, and polar aprotic solvent are versatile CO_2_ capture agents whose temperatures of carbonation and decarbonation are tunable over a wide range. The protic and aprotic polar solvents act as stabilizers and destabilizers, respectively, of the alkylcarbonate salt. Therefore, varying the solvent composition allows the carbonation and decarbonation temperatures to be optimized at different partial pressures of CO_2_. These findings provide valuable insights into the design and optimization of CO_2_ absorbents based on organic solutions.

## Results and discussion

Non-ionic mixtures of superbase and alcohol transform, as the carbonation progresses, into ion pairs consisting of a protonated superbase cation and an alkylcarbonate anion. They are of particular interest due to the large number of chemical combinations possible, the low temperature range of carbonation and decarbonation reactions, the new phase-switching or solid state modes of CO_2_ capture^18^,^28^,^29^.

**Figure 1:**
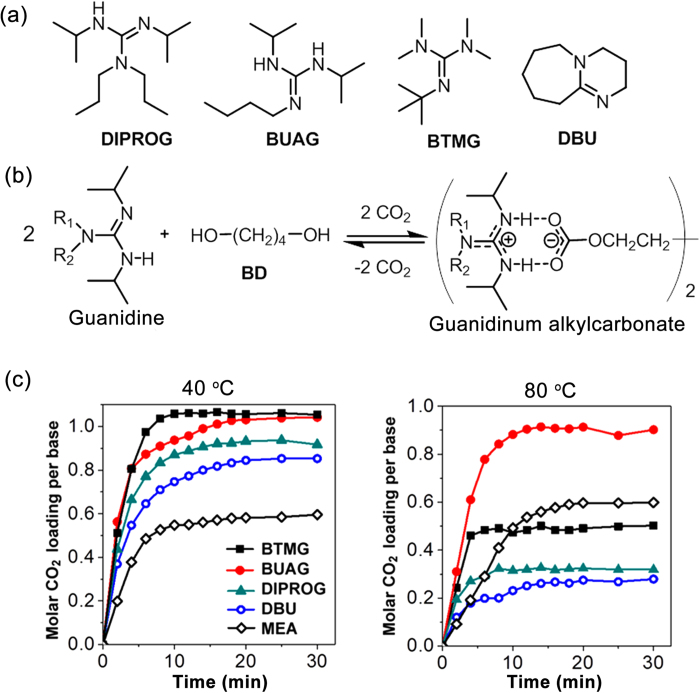
Carbonation of superbase/alcohol mixtures in a polar aprotic solvent. (**a**) The chemical structures of the superbases investigated are listed here and in the Supplementary Fig. 1. (**b**) Carbonation/decarbonation equilibrium of a guanidine/alcohol mixtures. (**c**) Molar CO_2_ loadings of the solutions of DIPROG, BUAG, BTMG, and DBU increased with time as CO_2_ was bubbled through the solution at a rate of 100 mL/min at 40 and 80 °C. The concentration of superbase was fixed at 40 wt% and the rest consisted of 1 eq. of BD relative to superbase, with NMP making up the remaining part. A 30 wt% aqueous solution of MEA was used for comparison.

We synthesized a number of substituted guanidines^30^ as listed in Fig. 1 and Supplementary Fig. 1, in which the guanidines containing one or two NH groups were categorized as type-I or type-II, respectively. Equivalent mixtures of guanidine and alcohol react with CO_2_ to yield guanidinium alkylcarbonate salts as confirmed by their NMR spectra (Supplementary Fig. 18 and 19). It has been known that a protonated guanidine and an alkylcarbonate anion form dual intermolecular hydrogen bonds^31^,^32^, as shown in Fig. 1b. Typical CO_2_ absorption curves with an equivalent mixture of superbase and BD dissolved in NMP at 40 °C and 80 °C are shown in Fig. 1c. The superbases compared were DIPROG (type-I), BUAG (type- II), 1,8-diazabicyclo[5.4.0]undec-7-ene (DBU), and 2-*tert*-butyl-1,1,3,3-tetramethylguanidine (BTMG), with a 30 wt% aqueous solution of 2-amino ethanol (MEA) tested for comparison^33^.

At 40 °C, with the same CO_2_ absorption setup and feed rate (Supplementary Fig. 17), the superbase solutions absorbed CO_2_ at higher rates and molar loadings than did the 30 wt% aqueous solution of MEA. At 80 °C, only BUAG absorbed CO_2_ about 0.9 mol while other superbases exhibited CO_2_ loadings below 0.5 mol. The MEA solutions showed no change in the CO_2_ loading at 80 °C.

**Figure 2:**
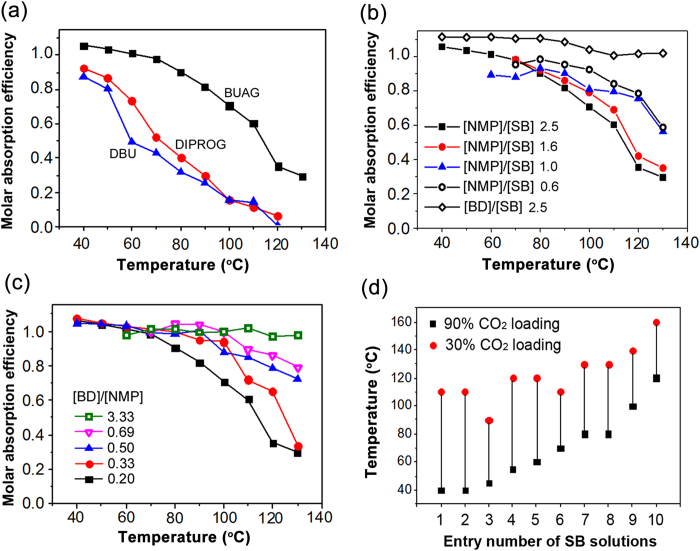
Equilibrium CO_2_ loading of the superbase solutions at various temperatures varying with the type of superbase and the polar protic/protic solvent compositions. (**a**) The mixtures consisting of superbase/BD/NMP (1:0.5:2.5), of which the superbase is BUAG, DIPROG, or DBU. (**b**) BUAG/BD mixtures (1:0.5 molar ratio) in solutions containing different amounts of NMP or BD (in molar ratio compared to BUAG). (**c**) BUAG solutions (40 wt%) with varying the molar ratios of BD and NMP. (**d**) The temperatures exhibiting 90 and 30 mol % CO_2_ loadings for 10 different solution mixtures of SB, BD, and NMP (detailed compositions in supplementary Table 1). Note that one mol of BD is equal to two equivalents.

We obtained equilibrium CO_2_ loadings (molar CO_2_ loading at saturation) for all solution mixtures from their absorption curves across the entire range of temperatures from 40 °C to 130 °C (Fig. 2 and Supplementary Fig. 20–25). We checked first if the stability of the alkylcarbonates was affected by the structure of superbase. Fig. 2a shows that, in a fixed molar ratio of superbase, BD, and NMP, the BUAG solution absorbed more CO_2_ than DIPROG or DBU solution in the entire range of temperature, indicating that the guanidines with more NH’s formed more stable alkylcarbonate salts than the other superbases. Similar trends were observed for other guanidine structures (Supplementary Fig. 24). The result is most likely caused by the hydrogen bonding ability of additional NH groups of type-II guanidines with the neighbouring carbonate ions in solution.

N-methyl-2-pyrrolidone (NMP), a polar aprotic solvent, appeared to be a good solvent for the guanidinium alkylcarbonate salts. We investigated how the amount of NMP added to the superbase/alcohol mixture affected the carbonation/decarbonation equilibrium (Fig. 2b). Equilibrium CO_2_ loading of the solution mixtures decreased as they were diluted with NMP. To lower the decarbonation temperatures, the solution needs to be composed of more polar aprotic solvent although dilution with solvent must be kept to a minimum to achieve the best gravimetric capture capacity. The solutions consisting of 40–60% superbase exhibited gravimetric capture capacities about 10 wt%.

In contrast to the effect of polar aprotic solvent, the superbase solution diluted with excess alcohol (protic solvent) maintained the fully saturated state even to temperatures greater than 130 °C. In Fig. 2b, the solutions of BUAG dissolved in BD of 5 equivalents (2.5 BD/SB molar ratio) shows no decarbonation at 130 °C. In general, an amount of alcoholic solvent greater than 2 eq. relative to BUAG resulted in decarbonation being difficult at temperatures below 100 °C.

Utilizing the opposite solvent effect of NMP and BD, the equilibrium CO_2_ loading of the solutions at elevated temperatures could be easily modified by adjusting the ratio of protic and aprotic solvents, as is clearly demonstrated in Fig. 2c, where equilibrium CO_2_ loading of 40 wt% BUAG solutions versus the solution temperature is plotted as a function of BD to NMP ratio. The data show that in the range of temperature greater than 70 °C the equilibrium CO_2_ loadings of the solutions is lowered by reducing the alcohol/NMP ratio of the solution. This means that the carbonation and decarbonation temperatures can be tuned in a wide range by varying the type and composition of superbase/alcohol/NMP mixtures as shown in Fig. 2d.

It is noteworthy that it was difficult to achieve full decarbonation of the guanidine solutions even at the high temperature region near 130 °C, where about 20–30 mol % residual carbonated species remained in the solutions even after heating for a few hours. These suggest that the stability of the carbonate salts depends strongly on the degree of carbonation of the solution itself, which may also be accounted for by the solvent effect^34^. Free alcohols and superbases are released from the salt as decarbonation proceeds, and become good hydrogen bond donors. They therefore add to the total concentration of protic solvating species, strengthening the thermal stabilities of the remaining carbonate salts.

An excess of the alcohol can, in principle, affect the carbonation/decarbonation equilibrium in two ways. It could simply increase the concentration of alcohol, resulting in a shift in the equilibrium toward the formation of the alkylcarbonate salt with no change in equilibrium constant (following Le Chatelier’s principle). In addition, it could stabilize the ion pairs by hydrogen-bonding, resulting in a change in the equilibrium constant that favors the carbonated state. To clarify the result with respect to the two effects, it was necessary to measure the equilibrium constant between carbonation and decarbonation.

**Figure 3:**
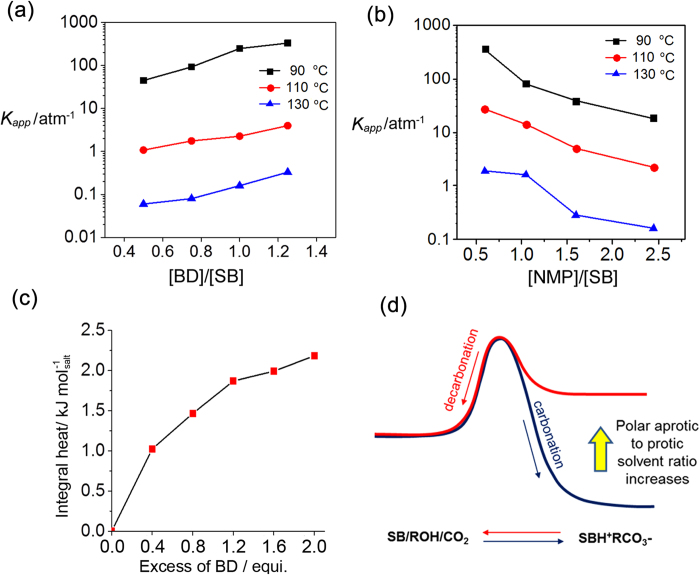
Solvent effect on stabilization and destabilization of guanidinium alkylcarbonate salts. ** a**) and **b**) Change in apparent equilibrium constant (*K*_*app*_) of carbonation with the molar ratio of BD and NMP, respectively, to a superbase(BUAG) at 90, 110, and 130 °C. **c**) Heat evolved upon titration of excess BD to a solution of a carbonated BUAG/BD salt (25 wt%) in NMP. **d**) Postulated free energy diagram of carbonation and decarbonation reaction varying with solvent composition.

We defined the apparent equilibrium constant (*K*_*app*_) as ([*SBH*^*+*^][*RCO*_*3*_^*−*^])/(*P*_*CO2*_ [*SB*][*ROH*]), where [*SBH*^*+*^], [*RCO*_*3*_^*−*^], [*SB*], and [*ROH*] are equivalent concentrations of guanidinium cation, alkylcarbonate anion, guanidine, and alcohol, respectively. By assuming the partial pressure of CO_2_ (*P*_*CO2*_) to be 1 atm, the values of *K*_*app*_ at different temperatures for each of the compositions were estimated from the absorption curves for BUAG. As shown in Fig. 3, *K*_*app*_ decreased with the concentration of NMP, but increased with that of BD, indicating an opposite effect of NMP and BD on the carbonation/decarbonation equilibrium. A similar trend of *K*_*app*_ for carbonation was observed for DIPROG, a type-I guanidine as shown in Supplementary Fig. 27. The change in equilibrium constants with solvent composition can be considered to be a direct indication of the effect of solvent on the stability of the alkylcarbonate salts.

Micro-calorimetric titration of the NMP solutions of the carbonated salts of an equivalent BUAG/BD mixture with excess BD (Supplementary Fig. 28) afforded the curve shown in Fig. 3c. It can be seen that approximately 2.0 kJ of heat per mole of superbase salt was evolved until approximately 2 eq. of excess BD was added to the solution of the salt. Although the heat cannot be considered to be precise solvation energy, it reflects that the carbonate anions unfavourably surrounded by NMP molecules became stabilized via hydrogen bonding of alcohol. It appears that approximately 2 eq. of hydroxylic groups were sufficient for obtaining efficient solvation of the carbonate anions. In Fig. 3d is illustrated how the polar aprotic to protic solvent ratio can alter the free energy of the carbonated state of the solutions at the elevated temperatures.

**Figure 4:**
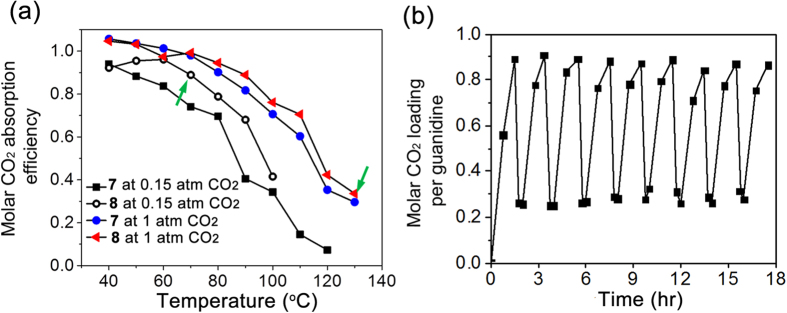
Carbonation and decarbonation at different partial pressures of CO_2_. **a**) Equilibrium CO_2_ loading of the solutions composed of BUAG/BD of 1:1 and 1:1.5, respectively, in the equivalent ratio (entry **7** and **8** in Supplementary Table 1) for CO_2_/N_2_ (15:85) mixed gas and pure CO_2_ of 1 atm. **b**) Carbonation and decarbonation cycle runs at the conditions marked with the arrows in Fig. 4a. The mixed gas was bubbled through the solution at a rate of 100 mL/min for carbonation at 70 °C, with pure CO_2_ employed at a rate of 10 mL/min for decarbonation at 130 °C.

The solution compositions may be tuned for carbonation and decarbonation at different partial pressures of CO_2_ that may be encountered in practical carbon capture applications. BUAG was employed to absorb CO_2_ from a mixed gas consisting of CO_2_ and N_2_ (15:85). For a mixture containing a stoichiometric BUAG/BD mixture (50 wt%) in NMP (entry **7** in Supplementary Table 1), the curve obtained for CO_2_ absorption from the mixed gas (*P*_*CO2*_ of 0.15 atm) was shifted towards lower absorption efficiency compared to that for pure 1 atm CO_2_ (Fig. 4a). To compensate the efficiency loss at the lower partial pressure the BUAG/BD equivalent ratio was increased to 1:1.5 (entry **8** in Supplementary Table 1). Notably, the pressure of CO_2_ in the regeneration process is likely to be higher, potentially 1 atm. The decarbonation efficiency of **8**, therefore, was estimated by employing the equilibrium CO_2_ loading curves for 1 atm CO_2_. The carbonation and decarbonation efficiency estimated using the efficiency-temperature curve of the solution **8** (Fig. 4a) indeed showed excellent agreement with those by cyclic runs of carbonation at 0.15 atm and decarbonation at 1 atm (Fig. 4b).

Tuning the carbonation and decarbonation temperatures allows the temperature gap between absorption and desorption to be minimized while maintaining the CO_2_ capture capacity, which may enhance the energy efficiency of carbon capture in technological application of the absorbents. In addition, facile tunability of the carbonation and decarbonation temperature may help to overcome the high viscosity problem of the organic sorbents. The viscosities of the fully carbonated solution (entry 7 and 8) in Fig. 4 were found to be 31 and 68 cP at 70 °C, as compared with 77, and 187 cP at 50 °C, respectively (Supplementary Fig. 30).

As a control, we studied the thermal stability of the BUAG/BD carbonate salt (50%) in n-hexadecane, a nonpolar solvent used in reference^27^. As a result, the mixture formed a homogenous solution at 130 °C but no decarbonation was observed (Supplementary Fig. 31). This is in contrast to the facilitated decarbonation in nonpolar medium. The difference may be accounted for by stronger hydrogen bonding between the guanidinium cation and alkylcarbonate anion pair in the present system. Nevertheless, it is clear that the stability of alkylcarbonate ion pairs in organic solutions cannot be controlled simply by altering the bulk polarity of solution.

## Conclusions

We have demonstrated that the thermal stabilities of guanidinium alkyl carbonates are readily tunable by varying the composition of superbase/protic solvent/polar aprotic solvent mixtures. Having more hydrogen-bondable components was found to stabilize the carbonated state; whereas, a polar aprotic solvent destabilized it. The high absorption rate, high capacity, reversibility, and superior chemical tunability of the guanidine/alcohol/NMP solutions are all highly attractive features for next-generation CO_2_ absorbents. Highly tunable characteristics will allow the absorbents to be used for effective purification of a variety of CO_2_-containing mixed gases, not only for the purpose of controlling CO_2_ emissions, but also for producing value-added gases by removing CO_2_ impurities under anhydrous conditions. The solvent effects on carbonation and decarbonation of the organic solution demonstrated here may be also utilized for creation of new switchable solvent or soft materials systems^28^.

## Methods

### Synthesis of guanidines

General Procedure: A mixture of amine (2eq.) and N,N’-diisopropylcarbodiimide (DIC) (1 eq.) were heated at 90–100 °C in a silicone oil bath for 5–7 h. The course of reaction was monitored by NMR analysis. After complete the reaction, excess amine was removed under reduced pressure. The crude reaction mixtures were distilled under reduced pressure to afford guanidines, yield >90%. Structure of the guanidines was confirmed by NMR spectroscopy.

### Typical procedure for synthesis of BUAG

A mixture of *n*-butylamine (23.1 g, 0.31 mol) and N,N’-diisopropylcarbodiimide (DIC) (20 g, 0.15 mol) were heated at 90 °C in a silicone oil bath for 5–7 h. Excess amine was then removed under reduced pressure. The resulting mixture was distilled under reduce pressure to yield colorless BUAG (b.p. 100 °C (0.5 mmHg); yield 96%).

### Carbonation and decarbonation of BUAG

The BUAG (5 g, 0.025 mol), 1,4-butanediol (1.13 g, 0.012 mol) , and NMP (6.1 g, 0.062 mol) mixtures were placed in a flask with a magnetic bar, and the flask was immersed in a pre-heated silicone oil bath to the appropriate temperature. The solution was stirred to produce a homogeneous solution prior to starting the absorption process. Following this, pure CO_2_ gas was bubbled through the solution using a stainless steel needle (100 mL/min.) The amount of absorbed CO_2_ was monitored at regular time intervals using an electronic balance with an accuracy of ±0.1 mg. The silicone oil on the exterior of the reaction flask was removed with n-hexane and the container was dried thoroughly before weighing. The other superbases such as DIPROG, DBU and BTMG were carbonated similarly. Decarbonation experiment was performed by heating with flowing CO_2_ at 10 ml/min at the desired temperature.

## Additional Information

**How to cite this article**: Rajamanickam, R. *et al.* Tuning Organic Carbon Dioxide Absorbents for Carbonation and Decarbonation. *Sci. Rep.* **5**, 10688; doi: 10.1038/srep10688 (2015).

## Supplementary Material

Supplementary Information
