# Supplementary material for: Tuning Organic Carbon Dioxide Absorbents for Carbonation and Decarbonation
Source: Sci Rep. 2015 Jun 2;5:10688. doi: 10.1038/srep10688 (PMC4451532; doi:10.1038/srep10688)
Supplement: Supplementary Information [file srep10688-s1.pdf]

Tuning Organic Carbon Dioxide Absorbents for Carbonation and Decarbonation

*Ramachandran Rajamanickam, Hyungsoo Kim, Ji-Woong Park**

School of Materials Science and Engineering, Gwangju Institute of Science and Technology, Gwangju, 500-712, Korea

Table of Content

**1**	Materials	**S3**
**2**	Synthesis of Guanidines	**S3**
**3**	Supplementary Scheme 1(Synthetic scheme of guanidine)	**S4**
**4**	Supplementary Figure 1(Structure of various guanidines)	**S4**
**5**	NMR spectra of guanidines	**S5**
**6**	CO_2 absorption experiment	**15**
**7**	Representative NMR spectra of guanidinum carbonate (BUAG-butanedicarbonate)	**S16**
**8**	Supplementary Table 1(Various superbases compositions)	**S18**
**9**	Supplementary carbonation and decarbonation data	**S19**

Materials

All amines and N,N-diisopropylcarbodiimide were purchased from Sigma-Aldrich and TCI chemicals (Korea) for the preparation of guanidines. Amines were distilled prior to use. 2-*tert*-Butyl-1,1,3,3-tetramethylguanidine (Barton's base), 1,8-Diazabicyclo[5.4.0]undec-7-ene (DBU), 1,4-butanediol, 1-hexanol, 2-aminoethanol (MEA) and N-methyl-2-pyrrolidone were purchased from Sigma-Aldrich and used as received. All other reagents and solvents were used of analytical grade. ^{1}H and ^{13}C NMR spectra were recorded on a Jeol-ECX-400P spectrophotometer using $CDCl_3$ or DMSO as solvent and Tetramethylsilane as internal standard. Chemical shift (δ) are given in parts per million (ppm).

General procedure for the synthesis of guanidines[1,2]

General procedure: A mixture of a mono-aminoalkane (2 eq.) and diisopropylcarbodiimide (1 eq.) were heated at 90-100 °C (for low boiling amines, 40-50°C) in silicon oil bath for 5-7 hours. The course of reaction was monitored by ^{1}H NMR analysis, when the reaction was completed; excess amine was removed by reduced pressure. The resultant crude mixture were distilled under reduced pressure to afford guanidines; Yield >90%. Syntheses of bifunctional guanidines are similar to the mono-functional guanidines, where excess carbodiimide (3 eq.) and 1eq. of diamine to be used to avoid the side products. The synthesized guanidines were confirmed by NMR analysis and their results are in accordance with earlier reports[1,2]. Guanidines such as PIPG, PROG, DIPROG, NMPG and BUAG were colorless liquids and MORG, CYCG and EDAG were obtained as white crystalline solid.

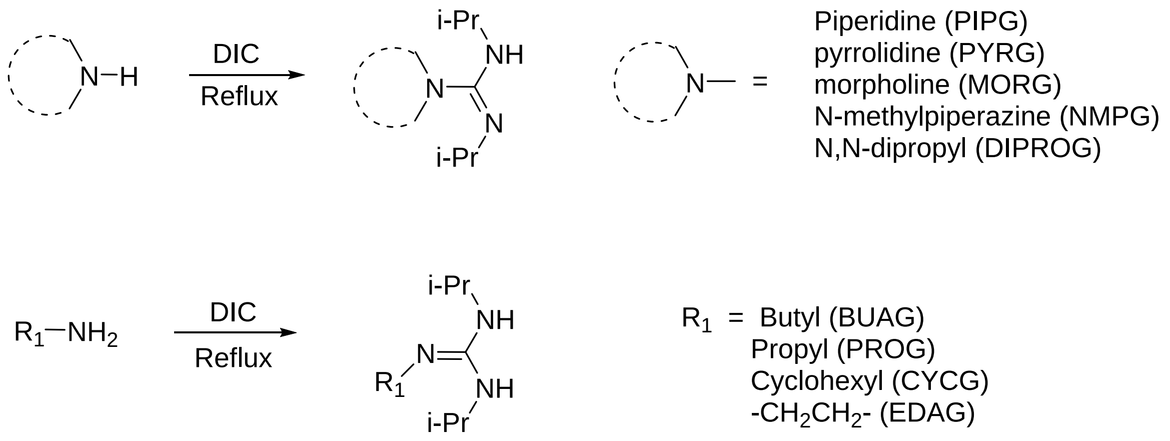

Supplementary Scheme 1. Synthesis of guanidines from amines and N,N'-diisopropylcarbodiimide.

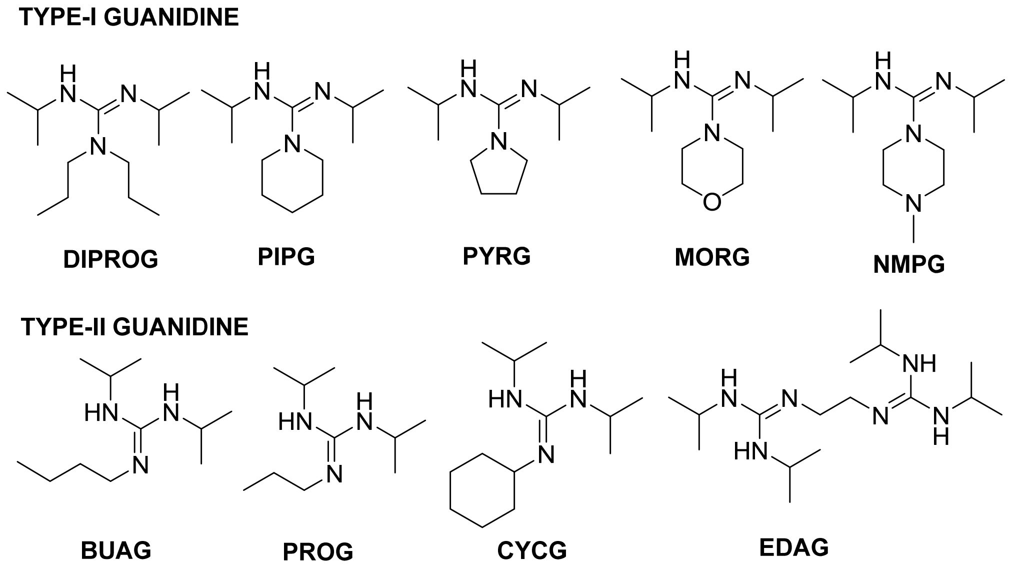

Supplementary Figure 1. Structure of various guanidines used for CO2 capture.

<u>NMR data:</u>

DIPROG: Colorless liquid, ^{1}H NMR, 400 MHz ($CDCl_3$, δ=ppm): 0.78 (t, 3H, CH_3,); 1.03 (q, 12H, CH_3 at isopropyl group); 1.44 (q, 4H, CH_2); 2.98 (q, 4H, CH_2); 3.26 (m, 1H, CH at isopropyl group); 3.39 (m, 1H, CH at isopropyl group). ^{13}CNMR, 100 MHz ($CDCl_3$, δ=ppm):11.64 (CH_3); 20.74 (CH_2); 23.79 & 24.94 (CH_3 at isopropyl group); 46.09 & 50.10 (CH); 47.44 (N-CH_2); 154.95 (C=N).

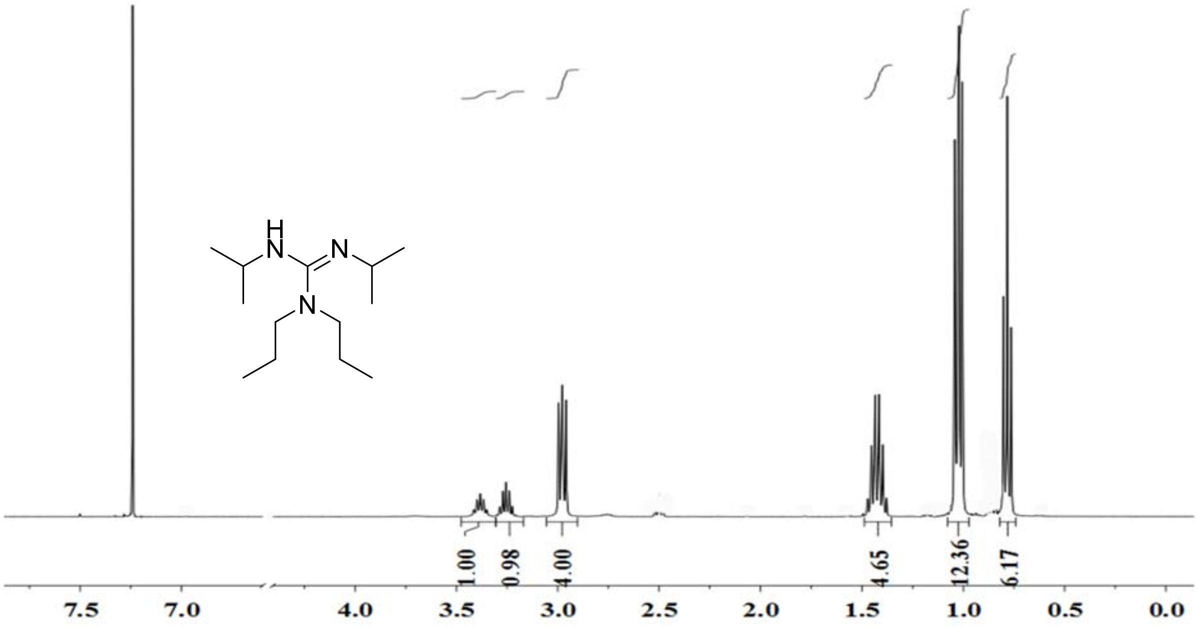

Supplementary Figure 2. ^{1}H NMR spectrum of DIPROG.

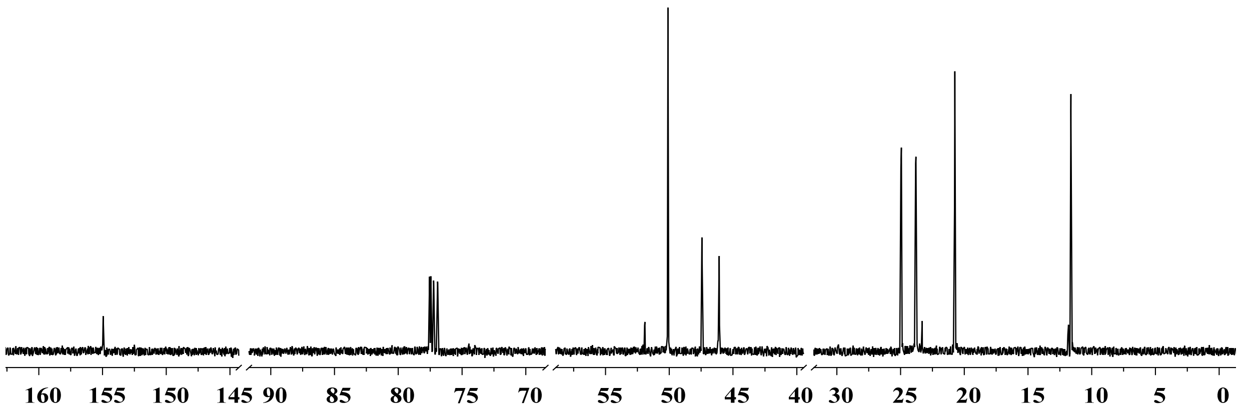

Supplementary Figure 3. ^{13}C NMR spectrum of DIPROG.

BUAG: Colorless liquid, ^{1}H NMR, 400MHz ($CDCl_3$, δ=ppm): 0.76 (t, 3H, CH_3); 0.96 (d, J=6.39Hz, 2H, CH_3); 1.21 (m, 2H, CH_2); 1.35 (m, 2H, CH_2); 2.84 (t, 2H, N-CH_2); 3.40 (bs, 2H, CH). ^{13}CNMR, 100MHz ($CDCl_3$, δ=ppm): 11.01 (CH_3); 13.82 (CH_2); 20.31 (CH_3); 23.66 (CH_2); 33.17 (CH_2); 43.72 & 44.18 (CH_2 & CH); 150.66 (C=N).

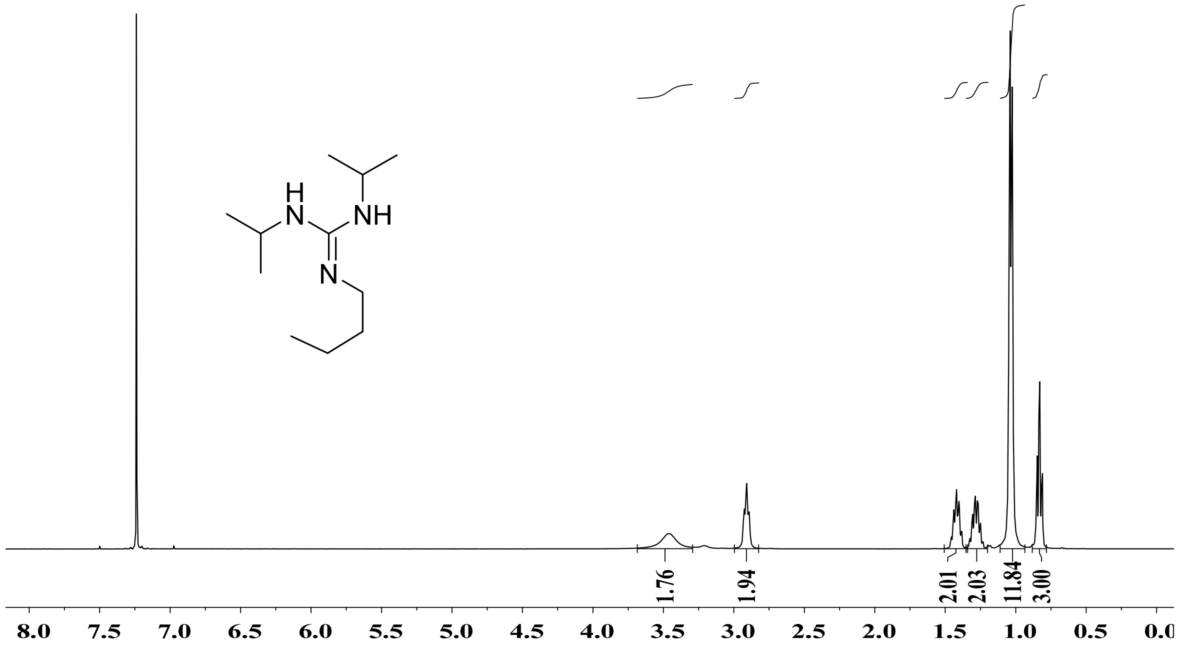

Supplementary Figure 4. ^{1}H NMR spectrum of BUAG.

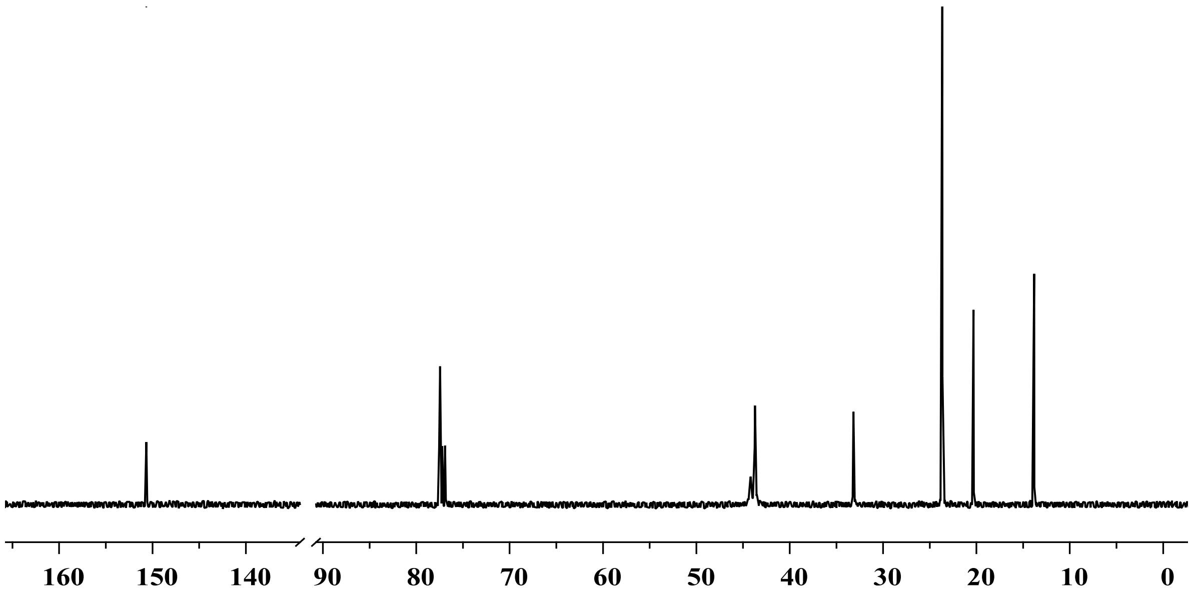

Supplementary Figure 5. ^{13}C NMR spectrum of BUAG.

PIPG: Colorless liquid, ^{1}H NMR, 400MHz ($CDCl_3$, δ=ppm): 1.01 (t, 12H, CH_3 at isopropyl group); 1.45 (bs, 6H, CH_2); 2.95 (bs, 4H, CH_2); 3.21 & 3.3 4 (m, 2H, CH at isopropyl). ^{13}CNMR, 100MHz, ($CDCl_3$, δ=ppm): 23.6 (CH_3); 24.9, 25.1, 26.1 (CH_2); 45.9 & 47.0 (CH) 49.0 (CH_2); 155.9 (C=N).

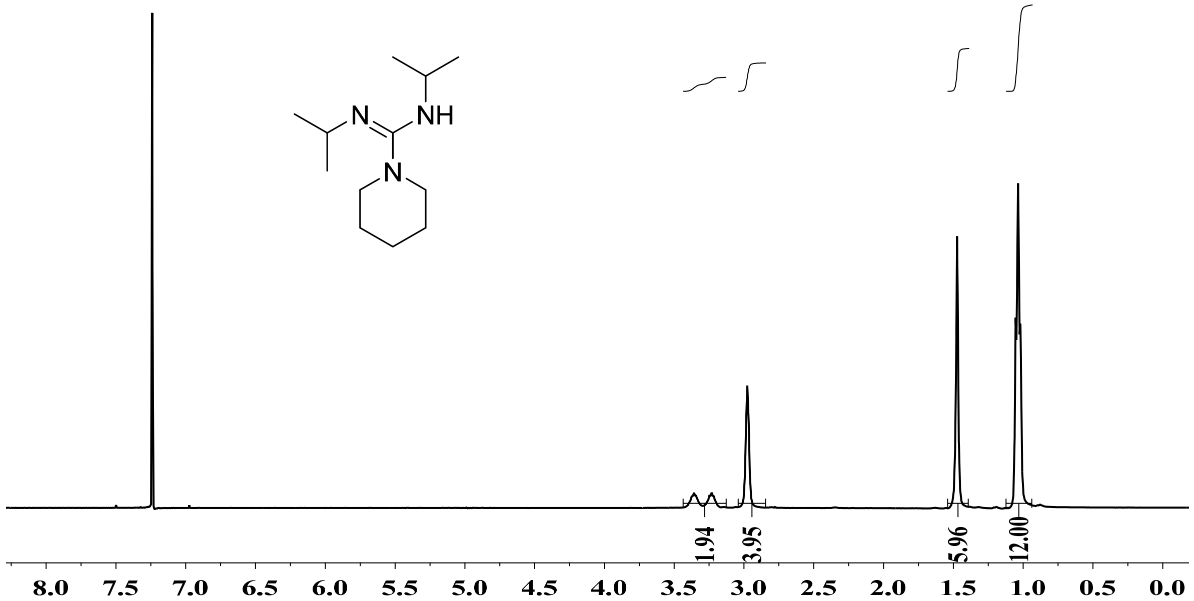

Supplementary Figure 6. ^{1}H NMR spectrum of PIPG.

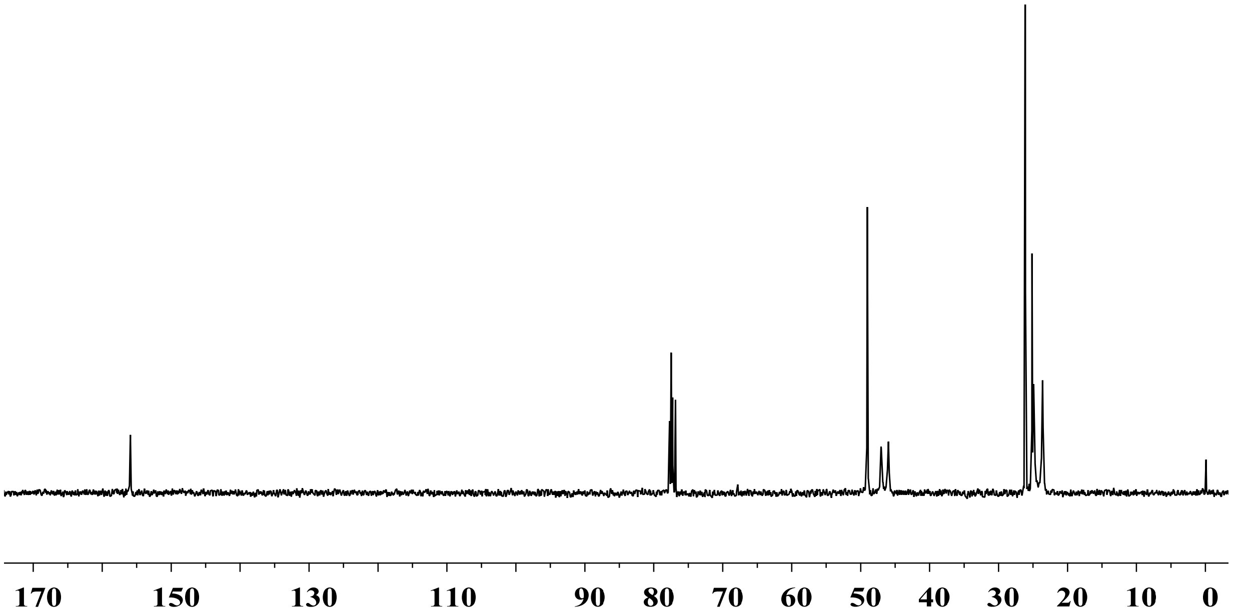

Supplementary Figure 7. ^{13}C NMR spectrum of PIPG.

PYRG: Colorless liquid, ^{1}H NMR ($CDCl_3$, δ=ppm): 1.02 (m, 12H CH_3 at isopropyl group); 1.72 (m, 4H, CH_2); 3.17(m, 4H, N-CH_2); 3.30 (bs, 2H, CH at isopropyl group group). ^{13}C NMR, 100 MHz, (DMSO, δ=ppm): 24.56 (CH_3); 24.35 & 45.63 (CH_2) 47.42 (CH); 152.32 (C=N).

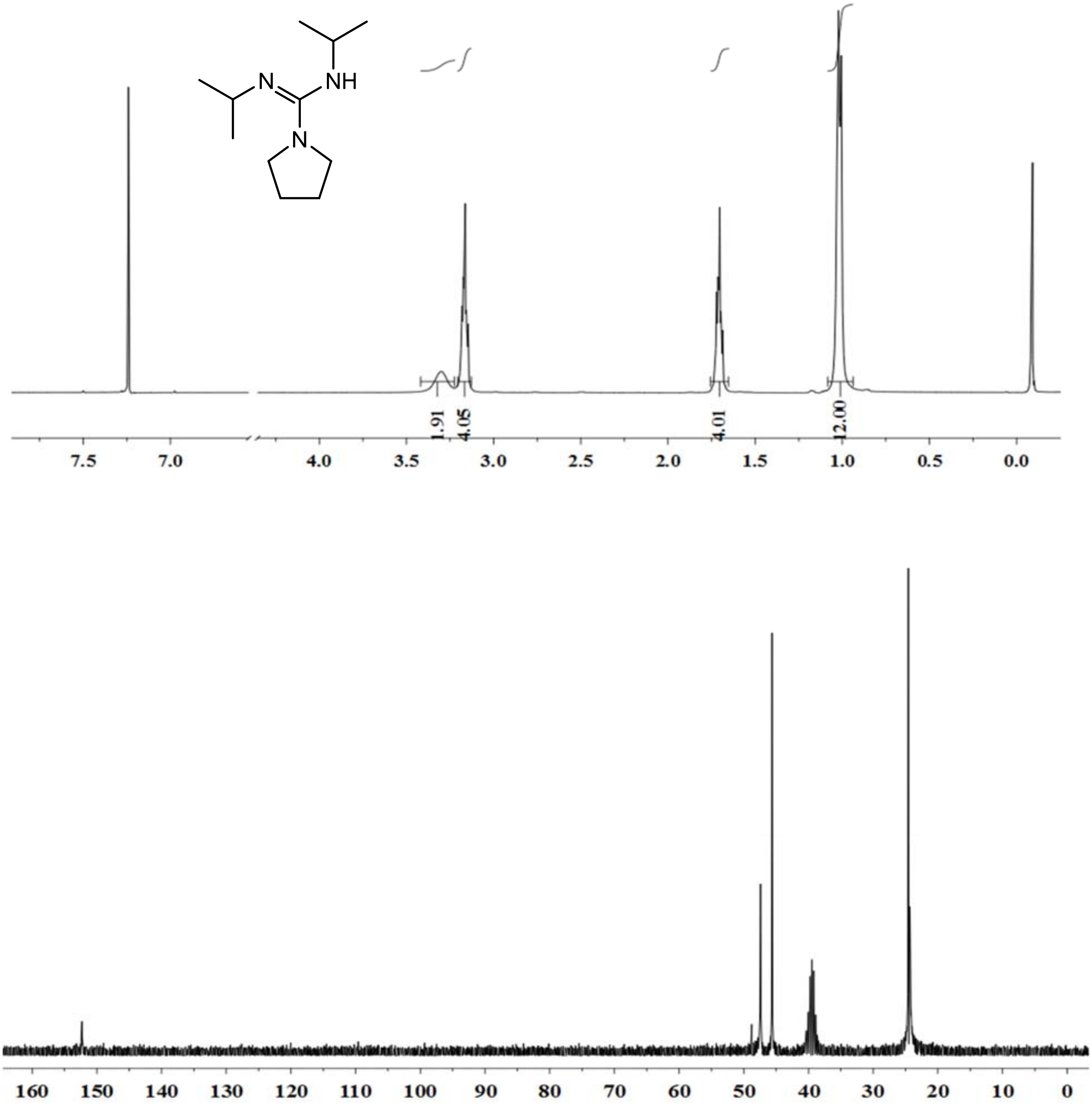

Supplementary Figure 8. ^{1}H(top) and ^{13}C(bottom) NMR spectrum of PYRG.

MORG: Colorless Solid, ^{1}H NMR, 400MHz, ($CDCl_3$, δ=ppm): 1.06 (dd, J=6.17 & 6.39 Hz, 12H CH_3 at isopropyl group); 3.04 (t, 4H, N-CH_2); 3.27 & 3.38 (m, 2H, CH at isopropyl group); 3.65(t, 4H, N-CH_2).

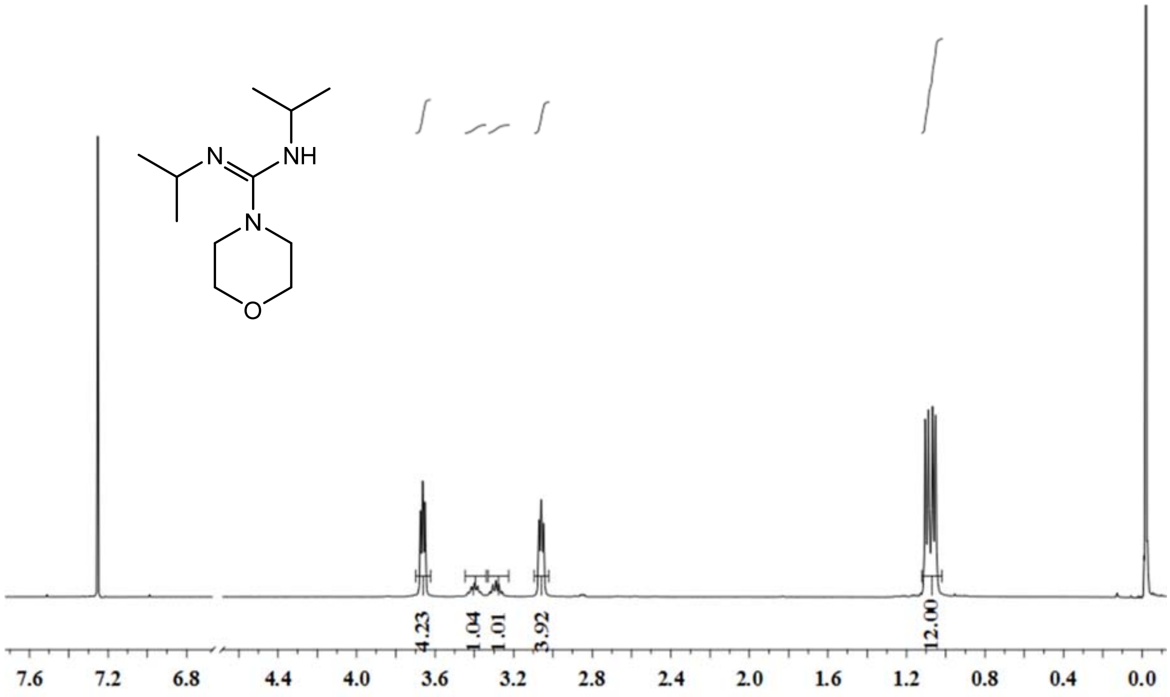

Supplementary Figure 9. ^{1}H NMR spectrum of MORG.

NMPG: Colorless liquid, ^{1}H NMR, 400MHz, ($CDCl_3$, δ=ppm): 0.94 (t, 12H CH_3 at isopropyl group); 2.14 (s, 3H, N-CH_3); 2.24, 2.97 (s, 8H, N-CH_2); 3.15, 3.24 (m, 2H, CH). ^{13}CNMR, 100MHz, ($CDCl_3$, δ=ppm): 23.3, 24.6 (CH_3); 46.24 & 46.67 (N-CH_2); 47.16 (CH); 55.0 (N-CH_3); 154.8 (C=N).

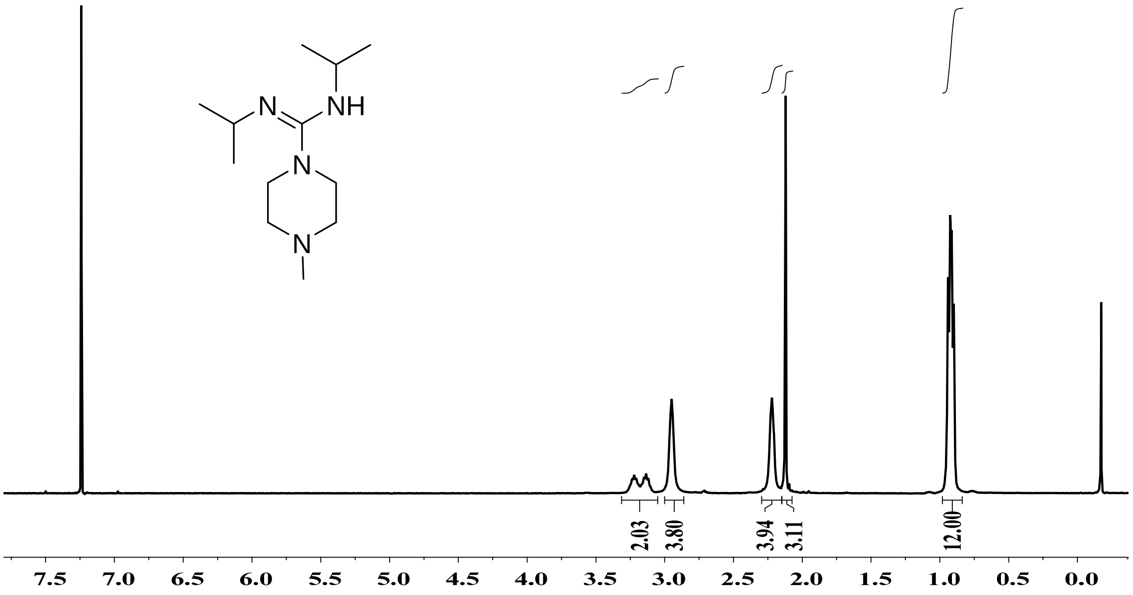

Supplementary Figure 10. ^{1}H NMR spectrum of NMPG.

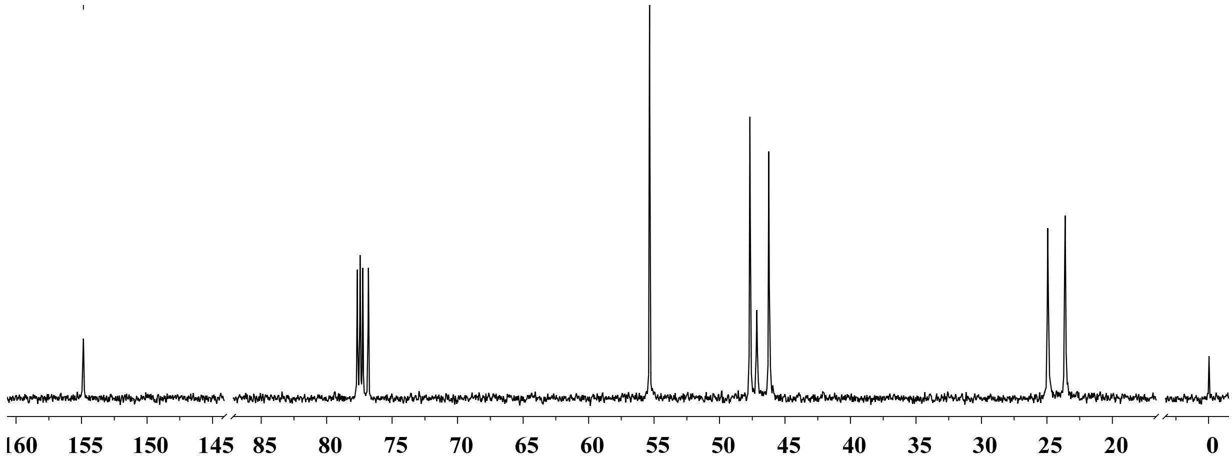

Supplementary Figure 11. ^{13}C NMR spectrum of NMPG.

CYCG: : Colorless solid, ^{1}H NMR, 400MHz ($CDCl_3$, δ=ppm): 1.083 (d, J=5.24, 12H, CH_3 at isopropyl group); 1.12-1.18 (m, 2H, CH2); 1.24-1.30 (m, 2H, CH_2), 1.57 (broad doublet, 1H, CH); 1.68 (broad doublet, 1H, CH); 1.83 (bs, 2H, CH2); 3.08 (bs, 1H, CH); 3.47 (bs, 2H). ^{13}C NMR, 100MHz ($CDCl_3$, δ=ppm): 23.95 (CH3 at isopropyl group); 25.19 (CH_2); 25.86 (CH_2); 34.45 (CH_2); 44.55 (CH at isopropyl group); 52.00 (CH at cyclohexane ring); 150.27 (C=N).

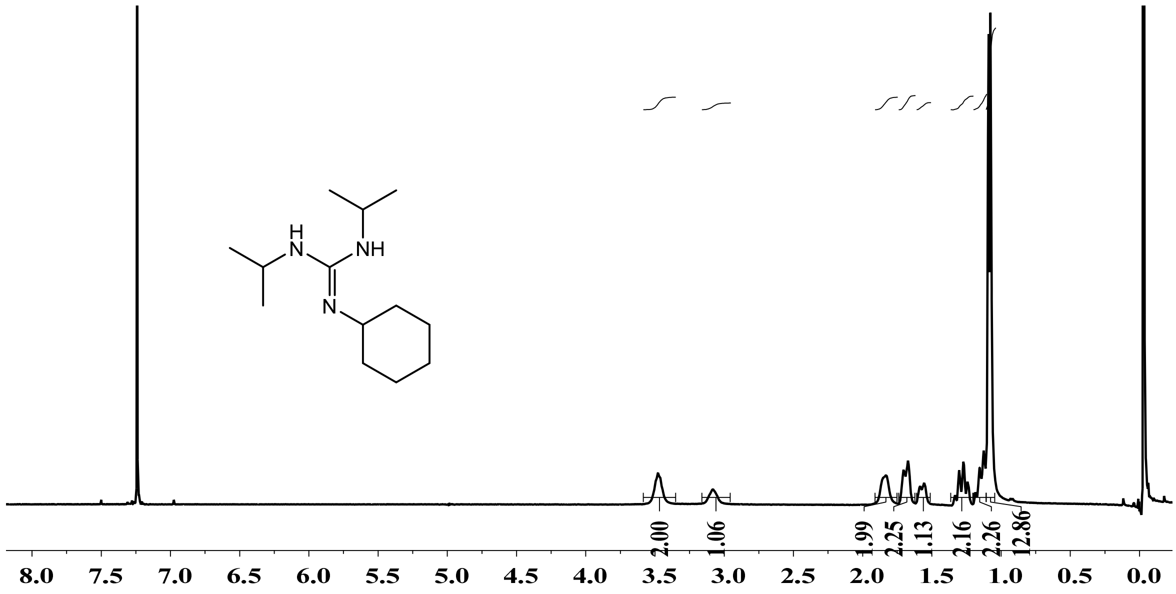

Supplementary Figure 12. ^{1}H NMR spectrum of CYCG.

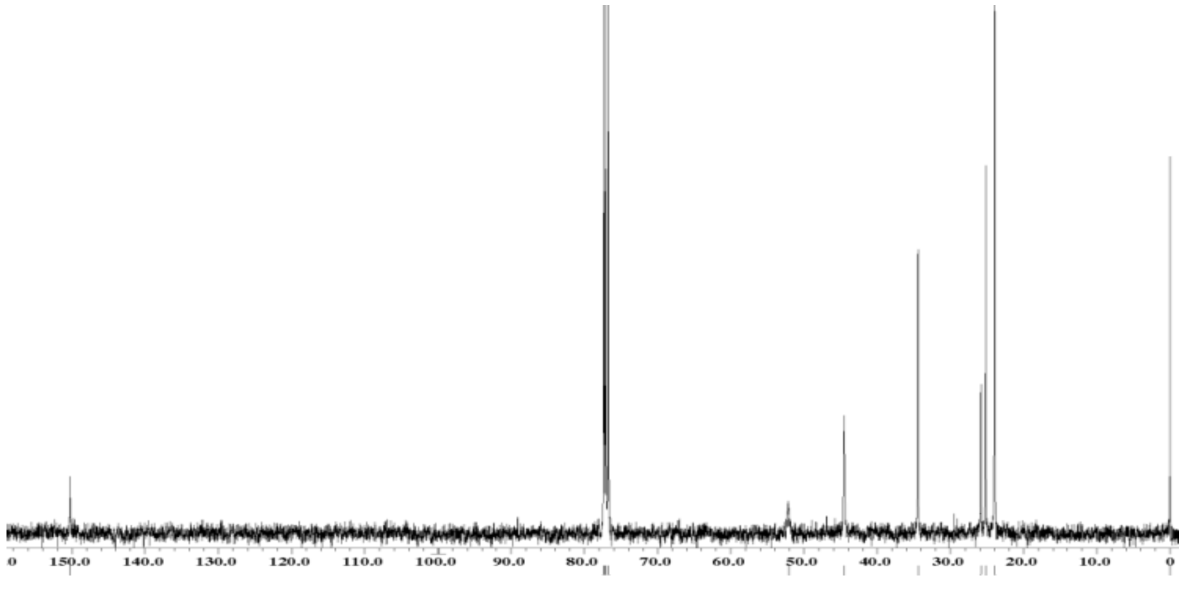

Supplementary Figure 13. ^{13}C NMR spectrum of CYCG.

EDAG: Colorless solid, ^{1}H NMR ($CDCl_3$, δ=ppm): 1.13 (t, 24H, CH_3 at isopropyl group); 3.14 (d, J=5.04, 4H, CH_2); 3.56 (m, 4H, CH at isopropyl group). ^{13}C NMR, 100 MHz, (DMSO, δ=ppm): 23.68 (CH_3); 42.49 (CH), 45.71 (CH_2); 150.77 (C=N).

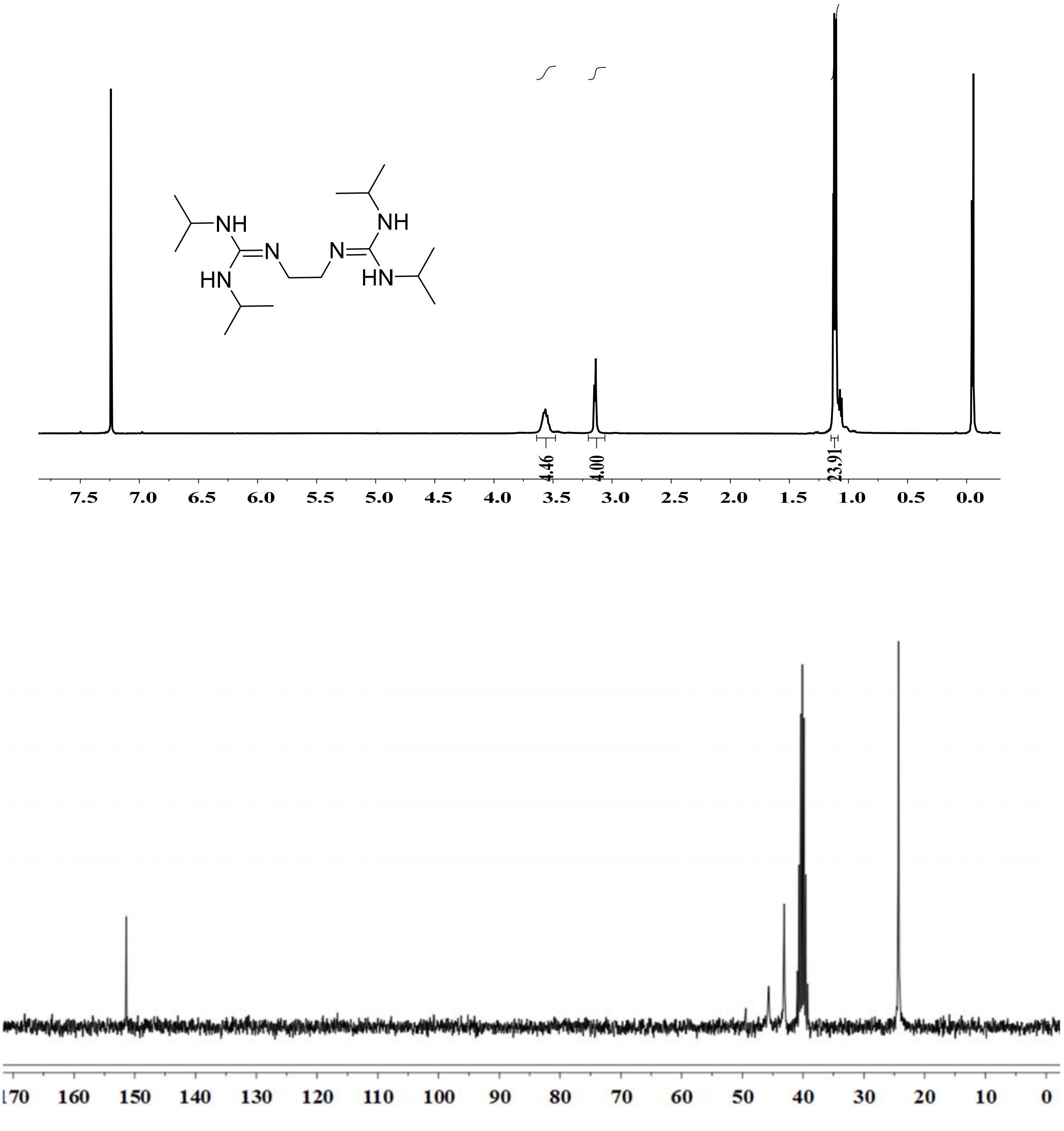

Supplementary Figure 14. ^{1}H(top) and ^{13}C(bottom) NMR spectrum of EDAG.

PROG: Colorless liquid, ^{1}H NMR, 400MHz, (DMSO, δ=ppm): 0.83(t, 3H CH_3at propyl group); .996 (d, J= 6.39, 12H, CH_3at isopropyl group); 1.40 (m, 2H, CH_2); 2.85 (t, 2H, CH_2); 3.57 (m, 2H, CH at isopropyl group). ^{13}C NMR, 100MHz ($CDCl_3$, δ=ppm): 11.8 (CH_3); 24.2 (-CH_3); 23.7 (CH_2); 43.8 (CH); 46.4 (CH_2) 150.7 (C=N).

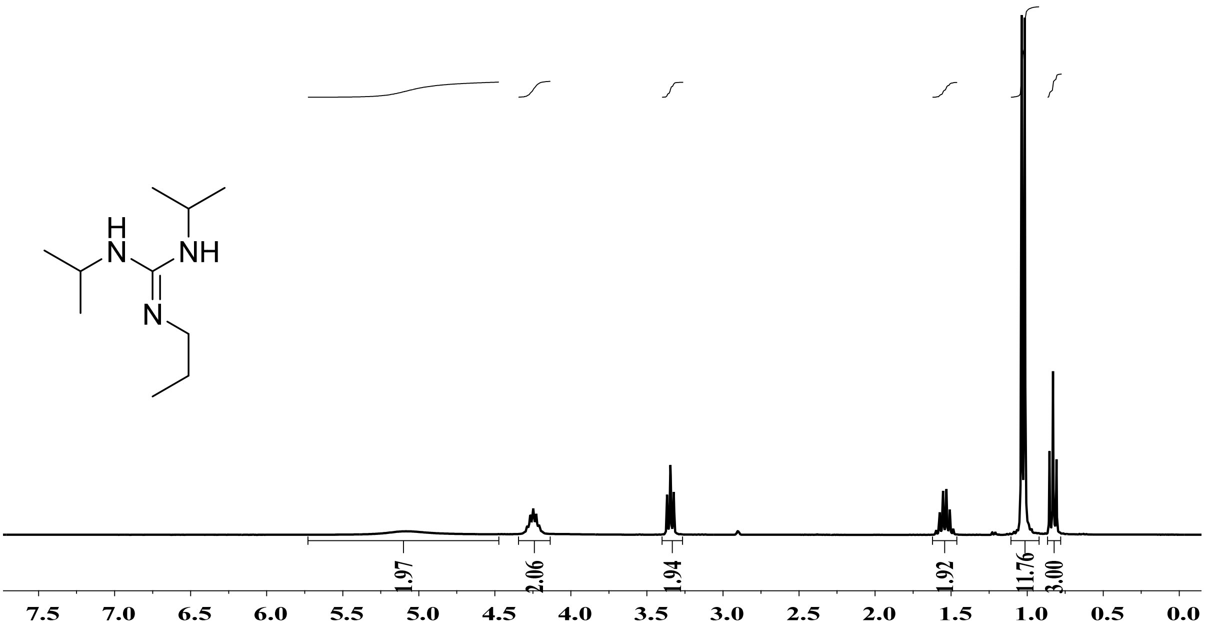

Supplementary Figure 15. ^{1}H NMR spectrum of PROG.

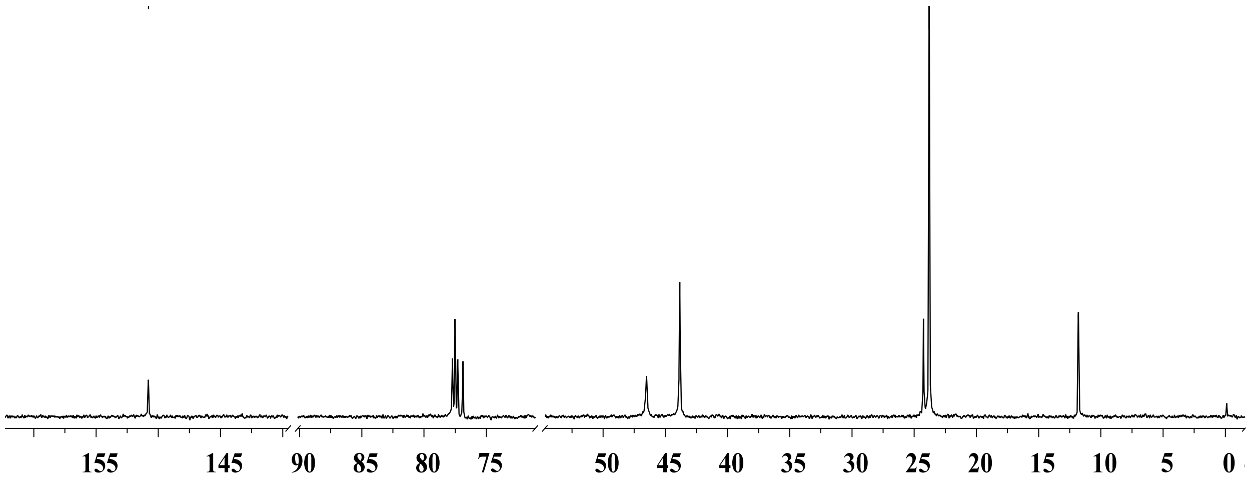

Supplementary Figure 16. ^{13}C NMR spectrum of PROG.

CO_2 Absorption Measurements:

The carbonation was performed by gravimetric method. The guanidines and 1,4-butanediol were degassed overnight prior to use. Pure CO_2 (1 atm, 99.9%) or N_2/CO_2 (85:15) mixed gas was used for carbonation experiment. The appropriate guanidine and alcohol mixture was put in a home-made three-neck flask equipped with a magnetic stirring bar and thermometer as shown in Supplementary Fig. 17. Then, the flask was immersed in a silicon oil bath pre-heated at the designated temperature. The mixture was stirred to get a homogeneous solution. Then, the CO_2 feed gas was bubbled through stainless steel needle (100 ml/min.) The amount of absorbed CO_2 was monitored for regular time intervals by measuring the weight change of the flask after removing the needle and condenser using electronic balance with an accuracy of ±0.1 mg. Silicon oil on the surface of the reaction flask was rinsed off by n-hexane before measurement. The 0.15 atm mixed gas (15% CO_2 and 85% N_2) experiment was carried out similar to the 1 atm CO_2 carbonation. Decarbonation experiment was performed by heating with flowing CO_2 at 10ml/min at the desired temperature.

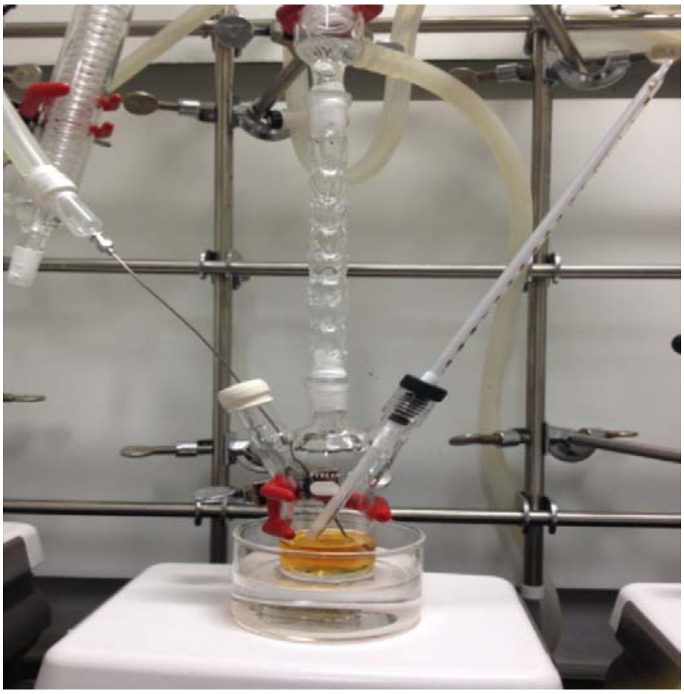

Supplementary Figure 17. Typical CO_2 capture experiment setup.

BUAG-1,4-butanedicarbonate

^{1}H NMR, 400MHz, (DMSO) δ = ppm: 0.86 (t, 3H, CH_3); 1.12 (d, J=8.00Hz, 12 H, CH_3 at isopropyl group); 1.26 (m, 2H, CH_2); 1.43 (m, 6H, CH_2); 3.19 (t, N-CH_2); 3.36 (m, 2H, CH_2); 3.61 (m, 2H, CH_2); 3.89 (m, 2H, CH). ^{13}C NMR, 100MHz, (DMSO) δ = ppm: 13.53 (CH_3), 19.31 (CH_2), 22.87 (CH_3-Isopropyl group), 26.32, 29.28 (CH_2), 30.80 (CH_2), 41.64 (N-CH_2), 43.59 (CH_2, CH), 60.67 & 63.53 (O-CH_2), 156.80 (C=N), 156. 80 & 159.73 (C=O).

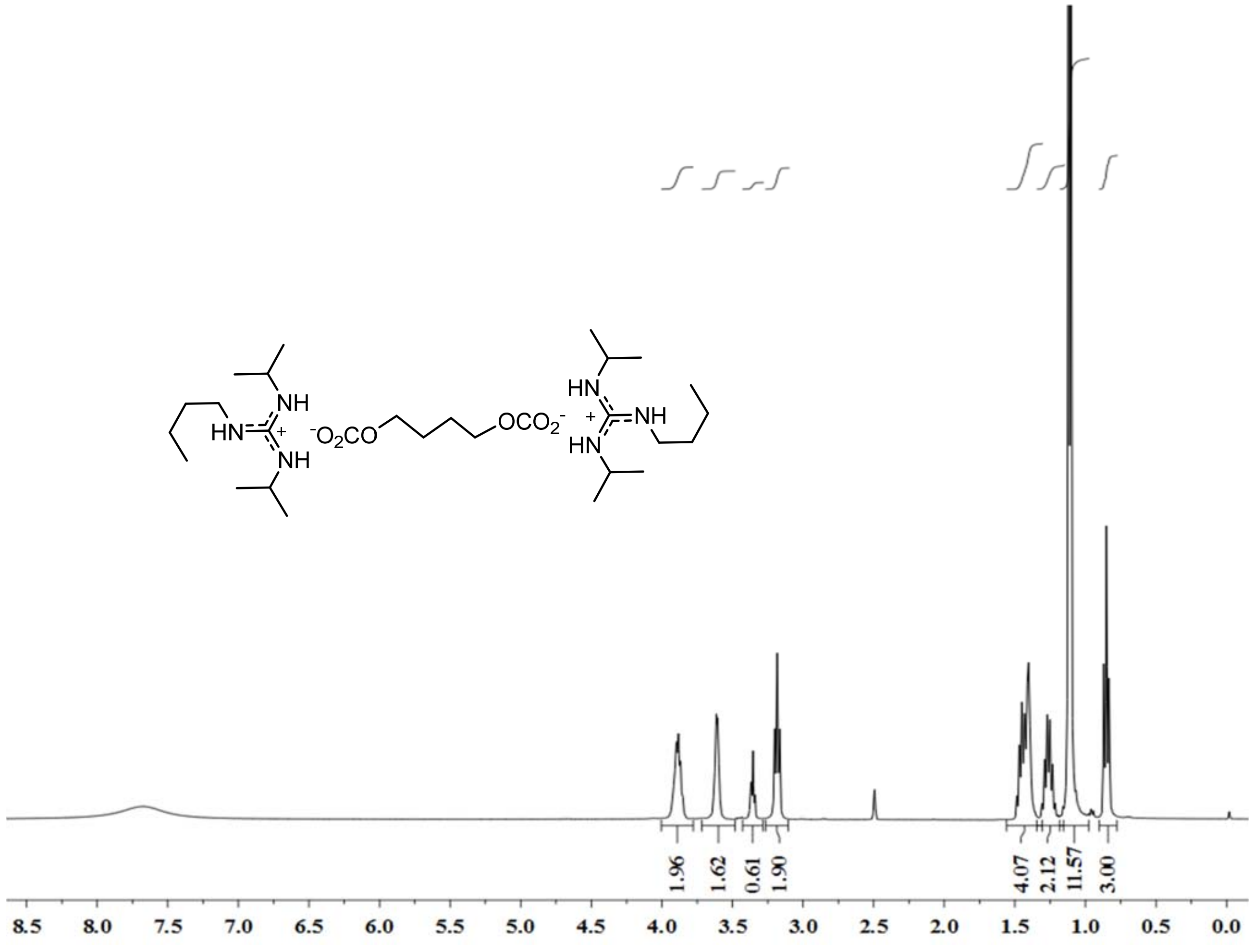

Supplementary Figure 18.^{1}H NMR spectrum of BUAG-1,4-butanedicarbonate.

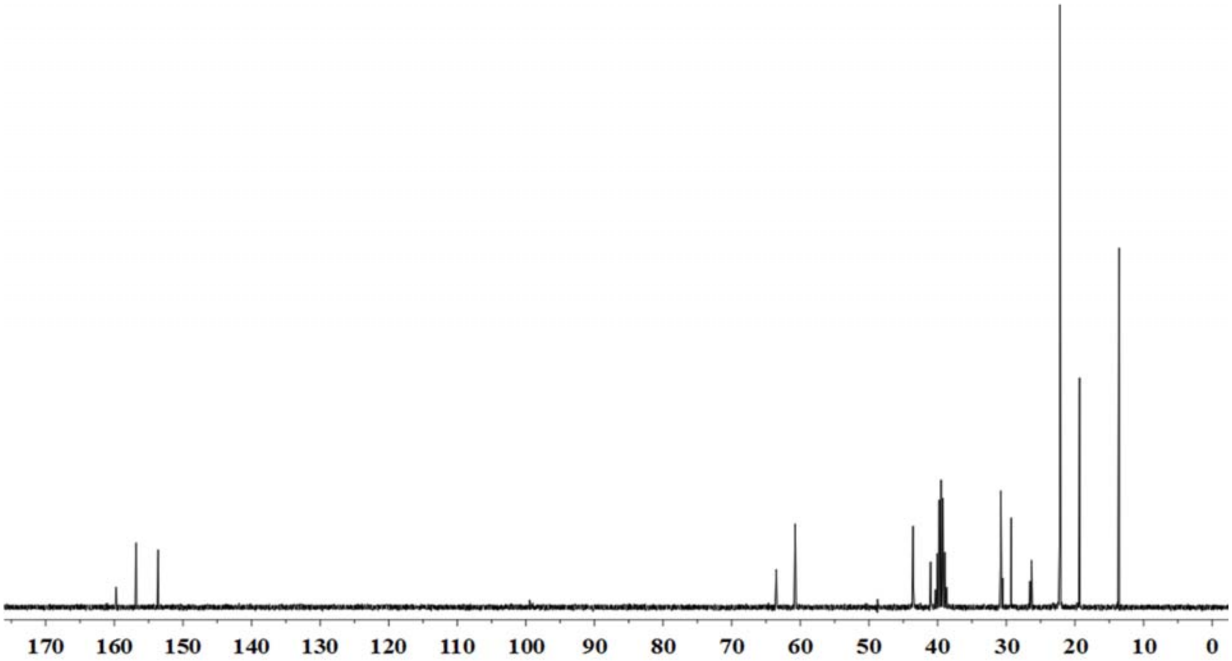

Supplementary Figure 19. ^{13}C NMR spectrum of BUAG-1,4-butanedicarbonate.

Supplementary Table 1. Ten Different superbase/alcohol/NMP compositions for Figure 2d.

Entry	Superbase used	molar ratio of SB/BD/NMP
**1**	DIPROG	1/0.5/2.8
**2**	DBU	1/0.5/2
**3**	PIPG	1/0.5/2.6
**4**	DIPROG	1/1/3
**5**	PIPG	1/1/2.8
**6**	DBU	1/1/3
**7**	BUAG	1/0.5/2.5
**8**	BUAG	1/0.75/2.7
**9**	BUAG	1/1/2.9
**10**	BUAG	1/2.5/4.3

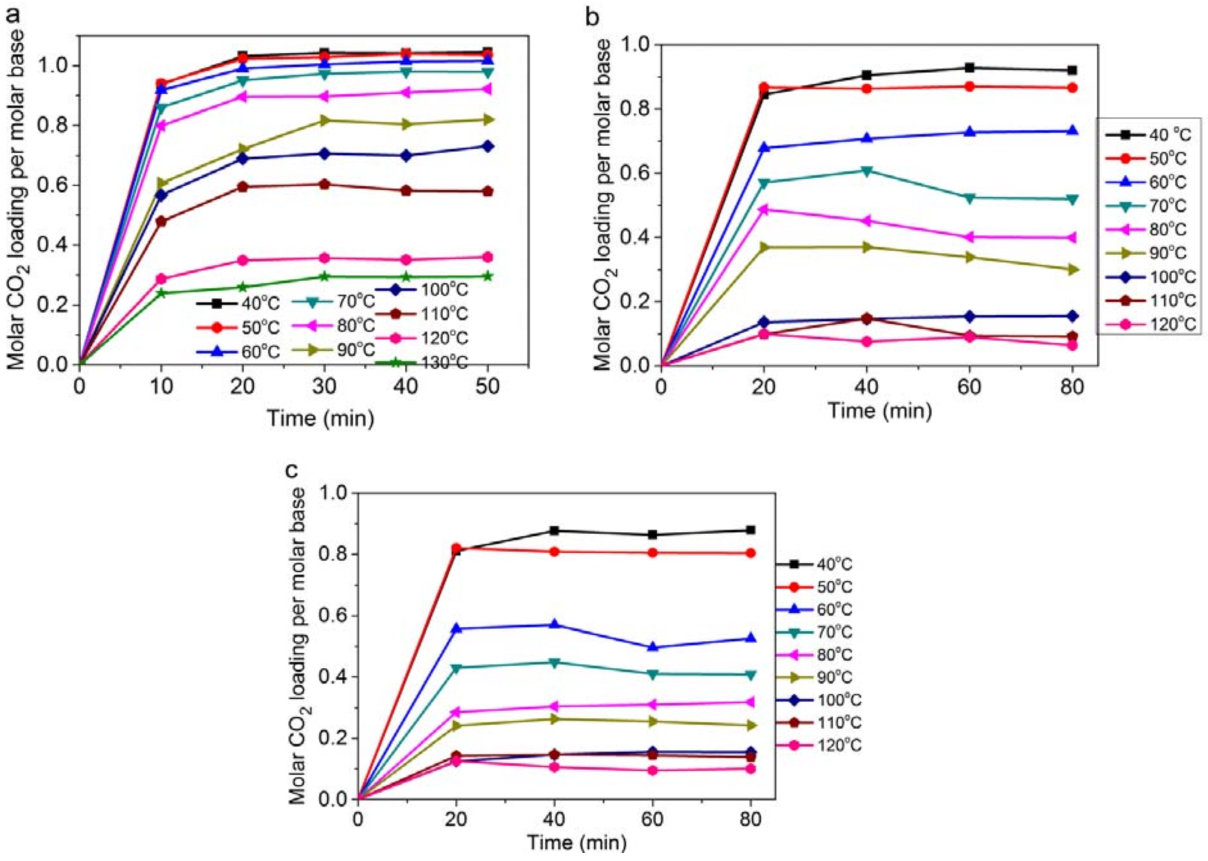

Supplementary Figure 20. CO_2 absorption curves at various temperatures obtained with varying the type of superbase: (a) BUAG, (b) DIPROG and (c) DBU with 1,4-butanediol and NMP in 1:0.5:2.5 (SB/BD/NMP) molar ratio. The 1 atm CO_2 was bubbled to the solution until saturated. We obtained the absorption curves in the range of 40 to 130°C.

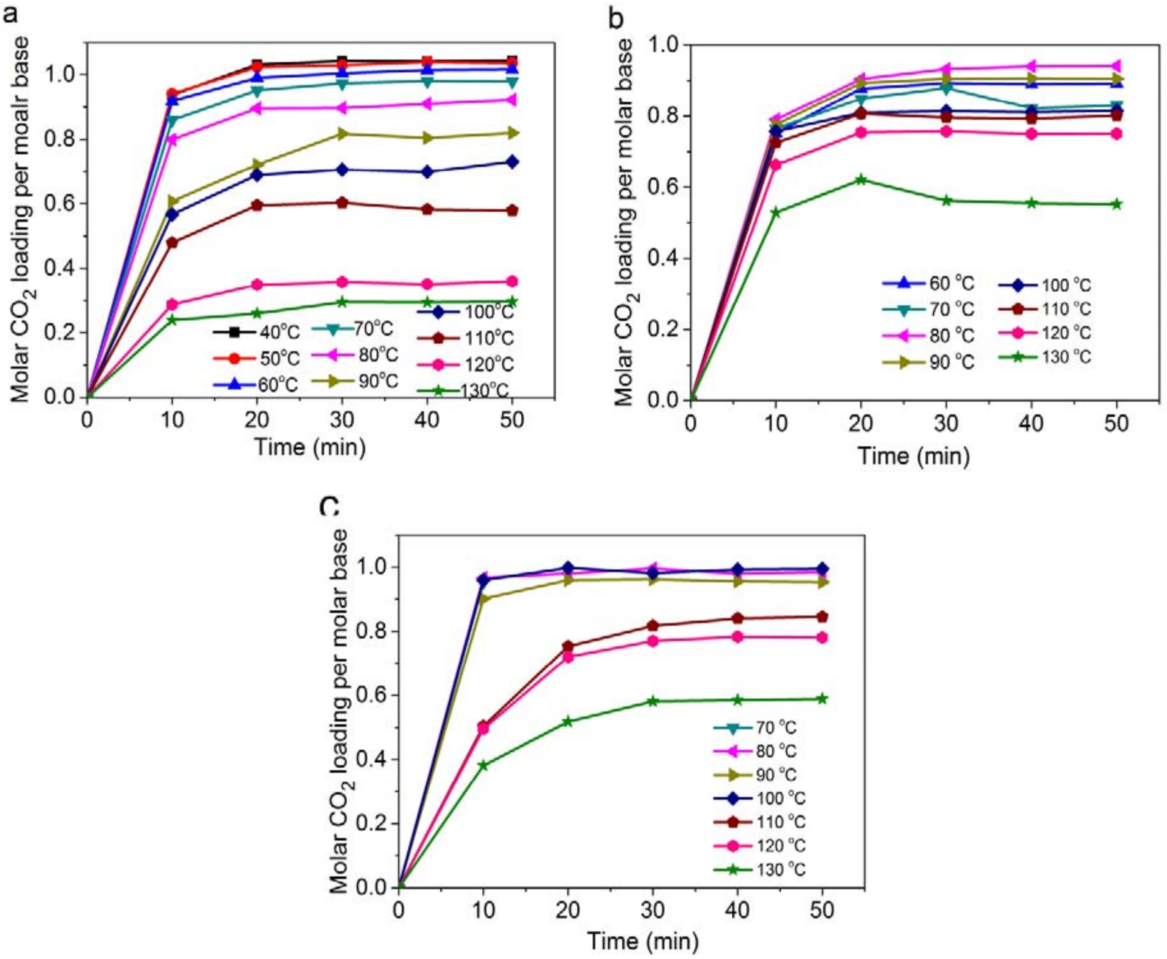

Supplementary Figure 21. CO_2 absorption curves obtained with varying the amount of polar aprotic solvent (NMP). The aprotic solvent ratio with respect to BUAG was varied while fixing the ratio of superbase (BUAG) to BD (1:0.5 molar ratio): (a) 2.5, (b) 1.0 and (c) 0.6 mol of NMP compared to BUAG.

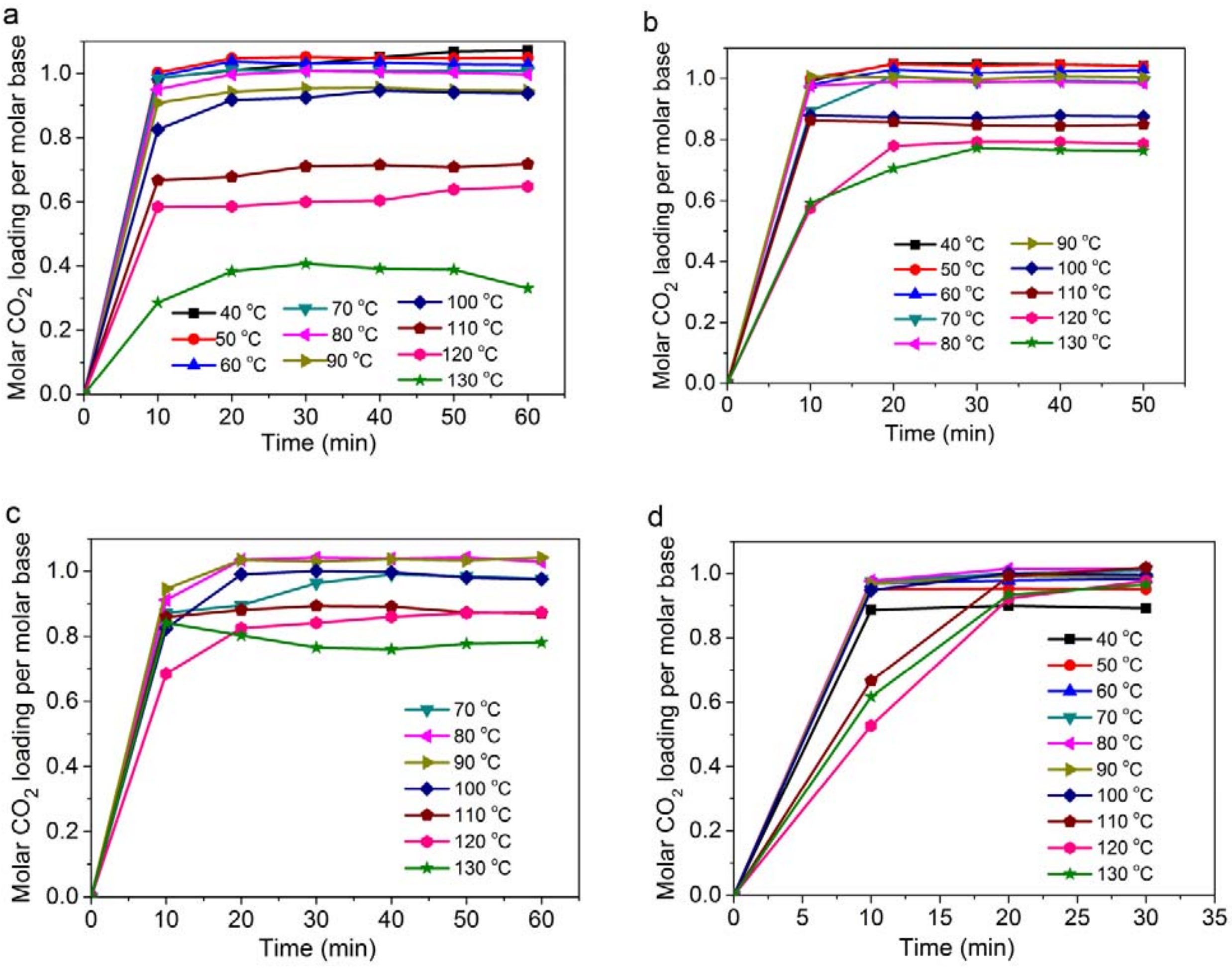

Supplementary Figure 22. The CO_2 absorption curves at various temperatures of a 40 wt% BUAG solution with the rest of the solution consisting of varying molar ratios of BD to NMP: (a) 0.69, (b) 0.50, (c) 0.33, and (d) 0.20.

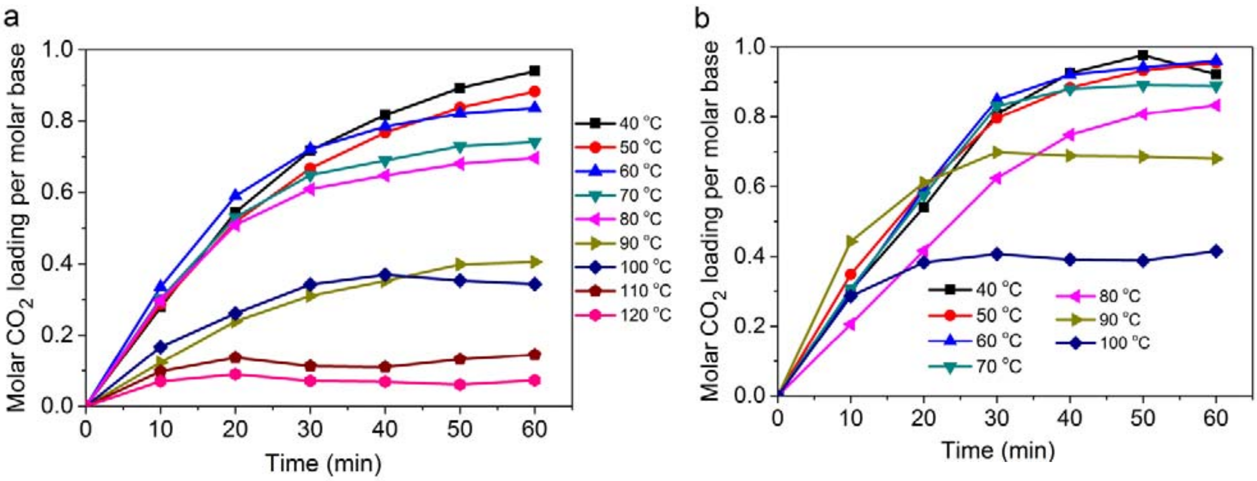

Supplementary Figure 23. CO_2 absorption curves of BUAG/BD/NMP mixtures for CO_2/N_2 (15/85) mixed gas at various temperatures. The mixed gas was bubbled through the solution at a rate of 100 mL/min. until the carbonation was saturated: BUAG/BD/NMP molar ratios are (a) 1/0.5/2.5 and (b) 1/0.75/2.7.

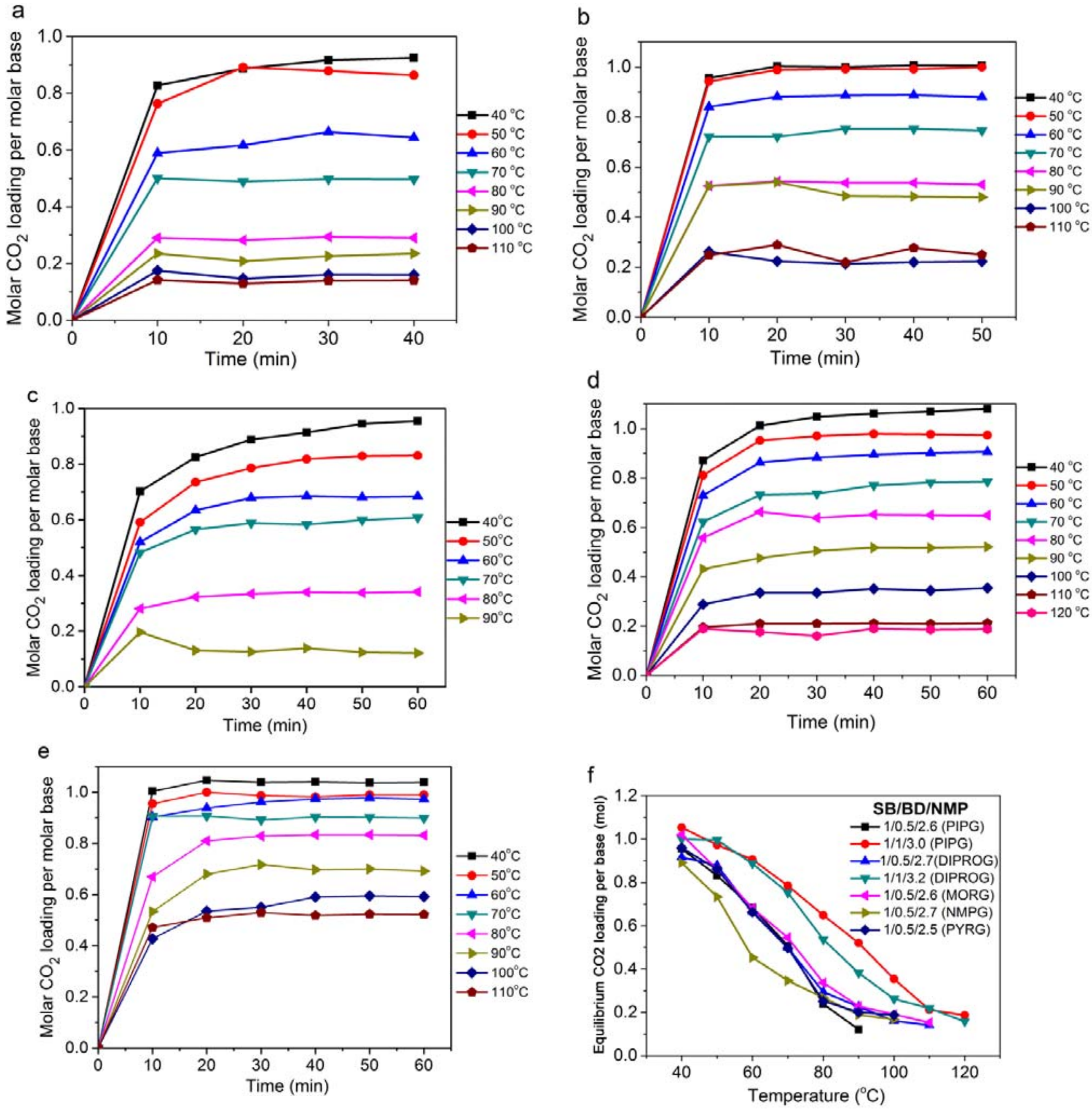

Supplementary Figure 24. (continued)

g

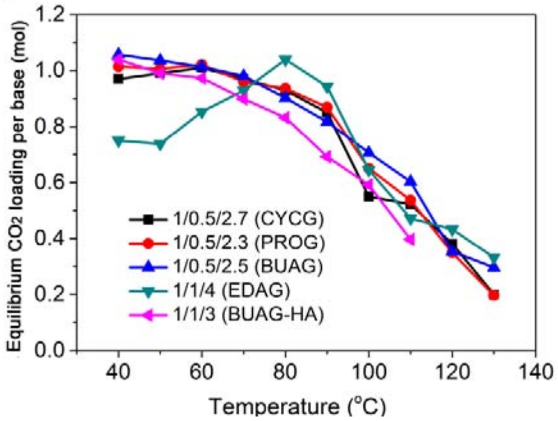

Supplementary Figure 24. CO_2 absorption curves for different superbase/alcohol/NMP mixtures. (a) DIPROG/BD/NMP (1/0.5/2.7), (b) DIPROG/BD/NMP (1/1/3.2), (c) PIPG/BD/NMP (1/0.5/2.6), (d) PIPG/BD/NMP (1/1/3.0), (e) BUAG/1-hexanol/NMP (1/1/3.0), (f) equilibrium CO_2 loading of all type-I guanidines at various temperatures, (g) equilibrium CO2 loading of all type-II guanidines at various temperatures.

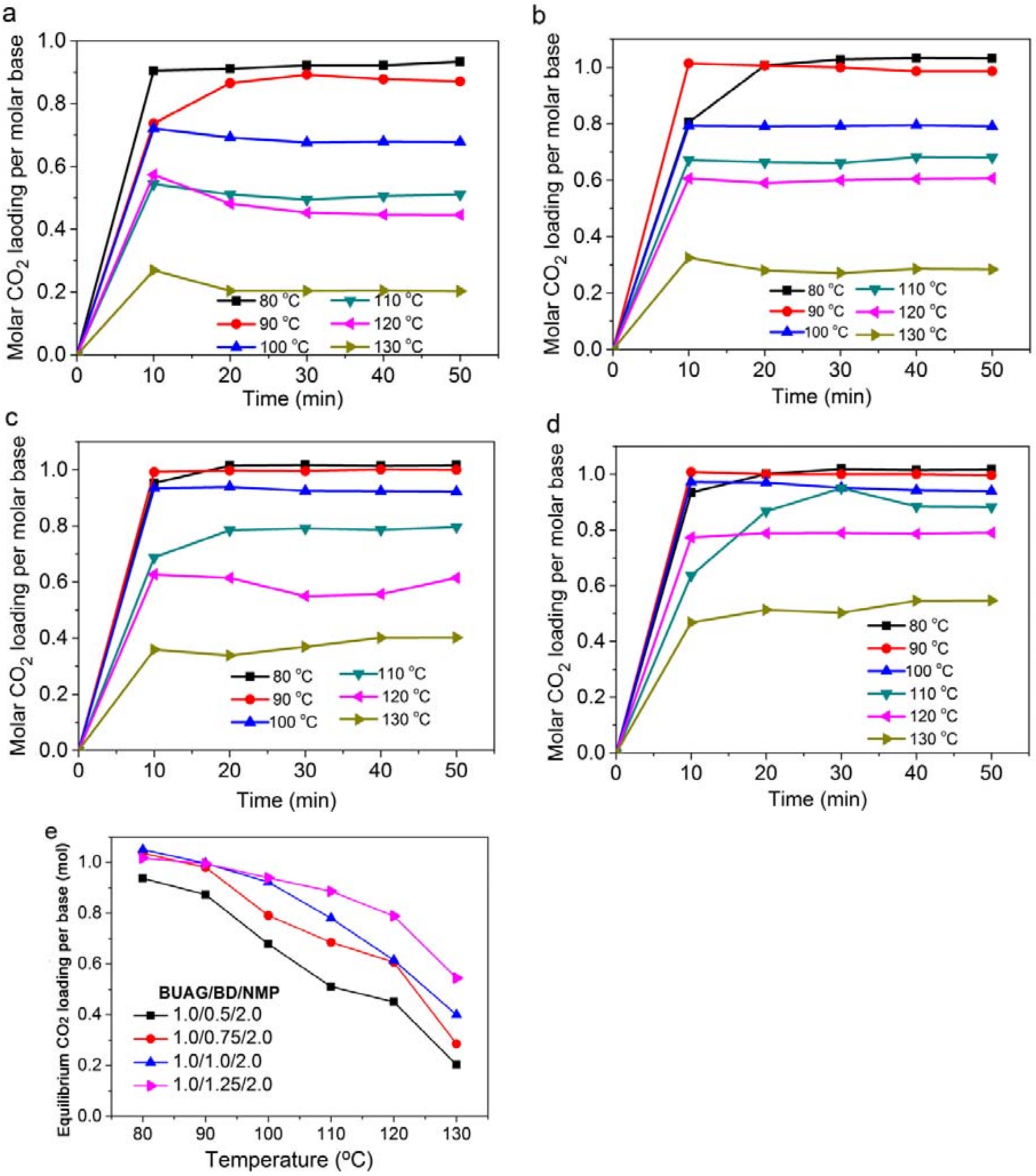

Supplementary Figure 25. CO_2 absorption by BUAG/BD/NMP mixtures with increasing the amount of BD while maintaining the 1:2 BUAG/NMP molar ratio. (a-d) CO_2 absorption curves at different temperatures in four different BUAG/BD/NMP ratios: (a) 1/0.5/2.0, (b) 1/0.75/2.0, (c) 1/1/2.0, and (d) 1/1.25/2.0. (e) Equilibrium molar CO_2 loadings for four compositions in the range of 80-130°C.

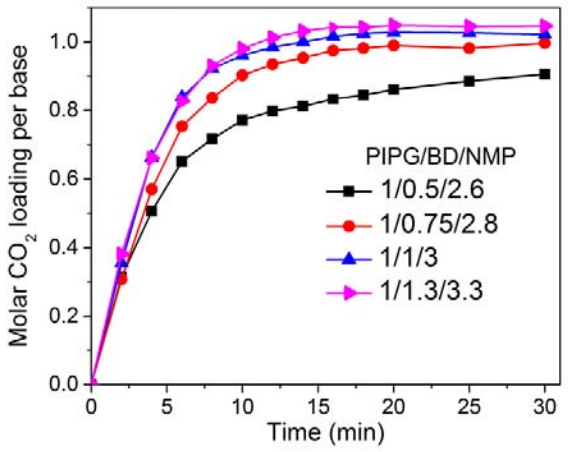

Supplementary Figure 26. Molar CO_2 absorption curves of PIPG/BD/NMP mixtures with increasing the ratio of BD to PIPG in 50% NMP solution at 40℃.

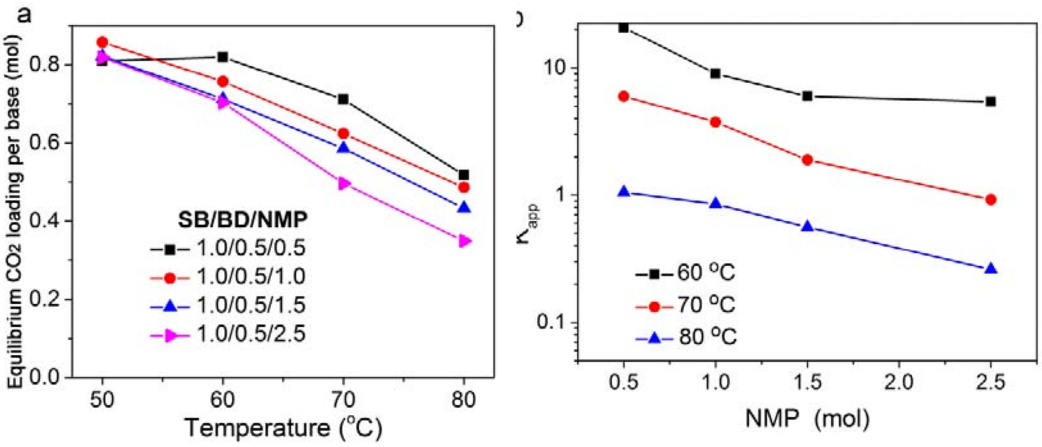

Supplementary Figure 27. (a) Effect of dilution with NMP on carbonation of DIPROG/BD (1/0.5 mol) at different temperatures. (b) Change in apparent equilibrium constant (K_{app}) of carbonation with different molar ratio of NMP to DIPROG.

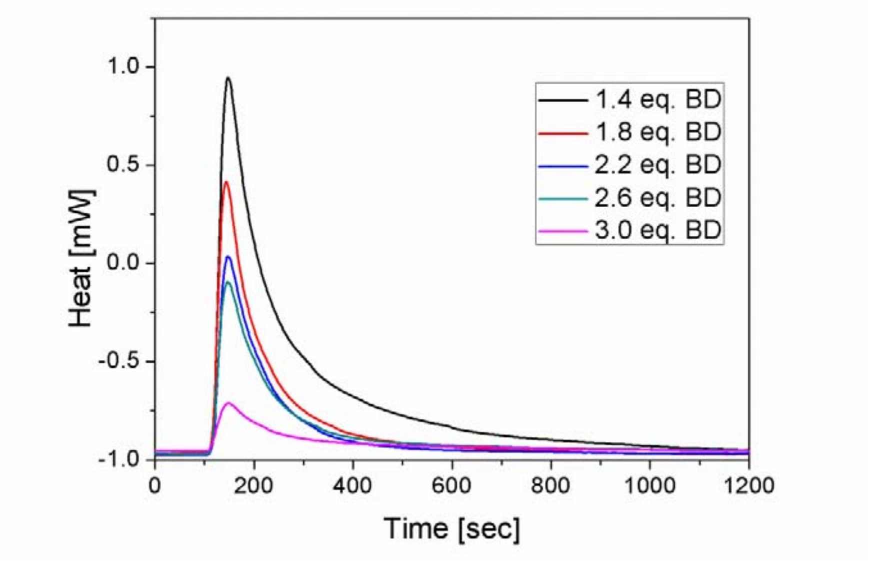

Supplementary Figure 28. Heat evolved from BUAG-BD carbonate (20 %) in NMP with increasing the equivalent number of BD at 25℃ on the microcalorimeter (Thermal Hazard Technology μRC-1.4.2).

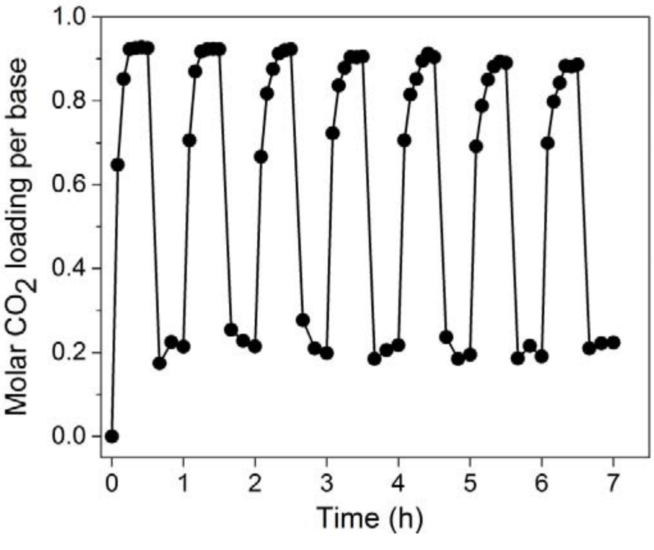

Supplementary Figure 29. Cyclic carbonation and decarbonation of BUAG/BD/NMP (1/0.5/2.5 molar ratio) with 1 atm CO_2 at 80 and 130℃, respectively. The experiment was carried out by flowing CO_2 at 1 atm in both carbonation and decarbonation. The results shows that the recyclability of this superbase (BUAG) solution was maintained their maximum carbonation efficiency constantly.

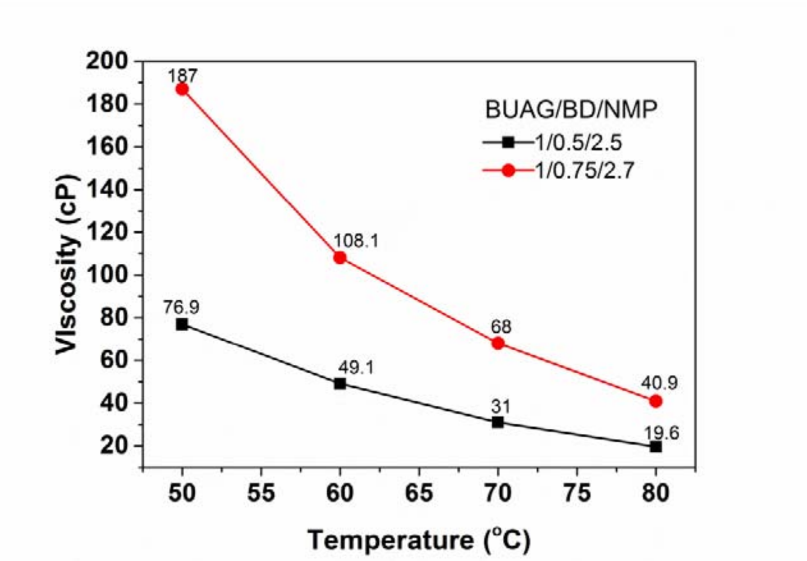

Supplementary Figure 30. Viscosity of two carbonated BUAG-BD-NMP mixtures (in molar ratio) at different temperatures. Viscosity was measured on Brookfield Viscometer LVDV-11+P CP.

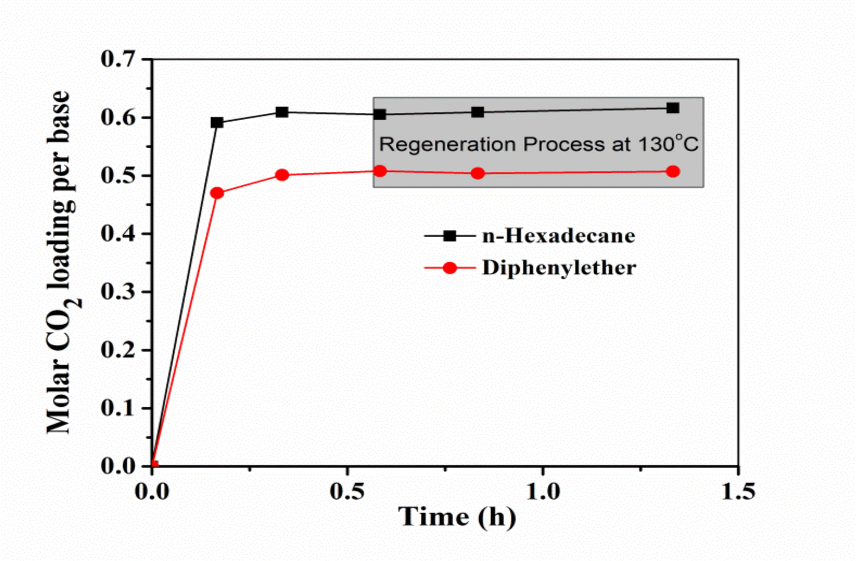

Supplementary Figure 31. Attempted decarbonation of BUAG-BD-CO2 complex in non-polar solvent (50wt %) at 130 ºC. The carbonate salt was obtained by performing absorption at 40 ºC.

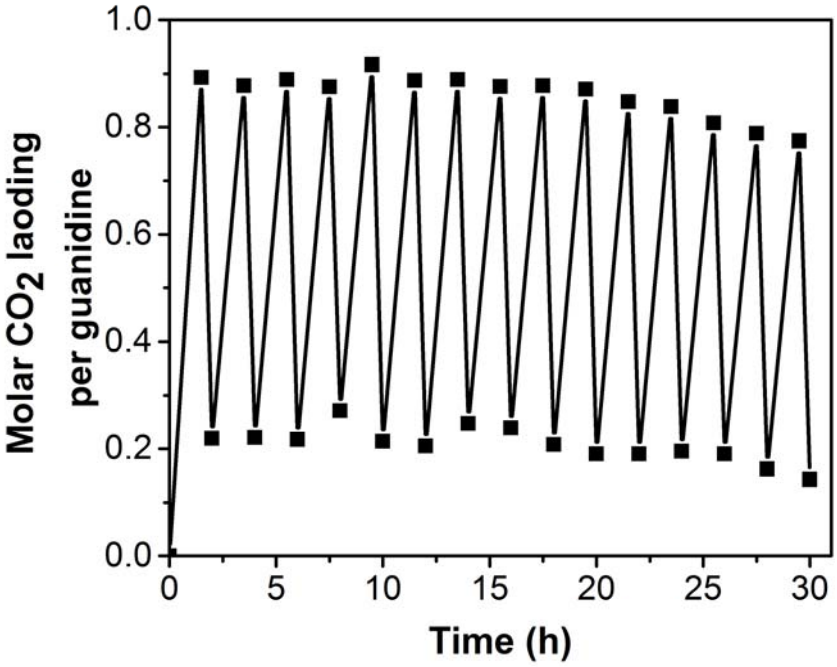

Supplementary Figure 32. Cyclic carbonation and decarbonation of BUAG/BD/NMP (1/0.75/2.5 molar ratio) with 0.15 atm CO_2 at 70 and 130℃, respectively. The experiment was carried out by flowing CO_2 at 1 atm in both carbonation and decarbonation. The results shows that the recyclability of this superbase (BUAG) solution was maintained their maximum carbonation efficiency constantly. However, the slight loss of weight in the efficiency is probably due to evaporation of NMP.

References

1. Shen, H.; Chan, H.-S. & Xie, Z. *Organometallics* **2006**, *25*, 5515.
2. Zhang, X. et al., *Tetrahedron* **2011**, *67*, 8790.
